# The Functions and Regulatory Mechanisms of Histone Modifications in Skeletal Muscle Development and Disease

**DOI:** 10.3390/ijms26083644

**Published:** 2025-04-12

**Authors:** Zining Huang, Linqing Hu, Zhiwei Liu, Shanshan Wang

**Affiliations:** 1 State Key Laboratory of Biocatalysis and Enzyme Engineering, National & Local Joint Engineering Research Center of High-Throughput Drug Screening Technology, School of Life Sciences, Hubei University, Wuhan 430062, China; huangzining@stu.hubu.edu.cn (Z.H.); linq.hu@stu.hubu.edu.cn (L.H.); 2 Key Laboratory of Swine Genetics and Breeding of the Ministry of Agriculture and Rural Affairs, Huazhong Agricultural University, Wuhan 430070, China

**Keywords:** skeletal muscle development, muscle disease, histone modifications

## Abstract

Skeletal muscle development is a complex biological process regulated by many factors, such as transcription factors, signaling pathways, and epigenetic modifications. Histone modifications are important epigenetic regulatory factors involved in various biological processes, including skeletal muscle development, and play a crucial role in the pathogenesis of skeletal muscle diseases. Histone modification regulators affect the expression of many genes involved in skeletal muscle development and disease by adding or removing certain chemical modifications. In this review, we comprehensively summarize the functions and regulatory activities of the histone modification regulators involved in skeletal muscle development, regeneration, and disease.

## 1. Introduction

Skeletal muscle is the most abundant tissue in the human body, accounting for over 40% of an adult’s body weight. It is indispensable for movement, gestural assistance, heat generation, protein storage, glucose and lipid homeostasis, and energy metabolism [1]. Therefore, appropriate muscle development is crucial for human health, mobility, and quality of life. The growth, development, and maintenance of skeletal muscle are highly organized processes that are tightly regulated by many factors. The dysregulation of these processes can lead to a variety of muscle disorders, including sarcopenia, cachexia, atrophy, and muscular dystrophies, and these diseases will seriously lower people’s living standards.

**Figure 1:**
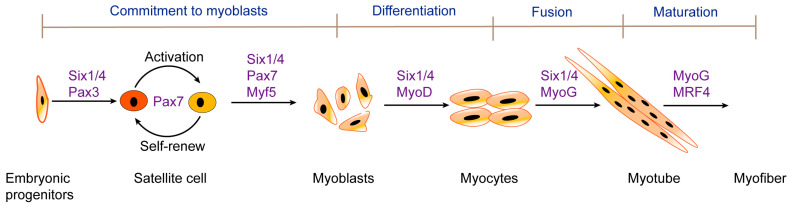
Expression profiles of transcription factors that regulate the progression of myogenic lineages during skeletal muscle development and regeneration. Six1/4 and Pax3/7 are the main regulatory factors of early lineage norms. Satellite cells expressing Pax7 are usually in a quiescent state and are activated to become myoblasts expressing Myf5 after muscle injury, with some satellite cells undergoing self-renewal to replenish the stem cell pool. The proliferating myoblasts express Myf5 and MyoD, while Pax7 is downregulated. MyoD is expressed in early differentiated myoblasts, and the expression of the terminal differentiation genes required for myotube and myofiber formation is jointly accomplished by myogenin (MyoG) and MRF4.

During embryogenesis, skeletal muscle originates from paraxial mesoderm, which segments into somites on either side of the neural tube and notochord [2]. Progenitor cells in somites give rise to the skeletal muscles of the body and the limbs of vertebrates [3]. These progenitor cells delaminate from the dermomyotome, which is the hypaxial edge of the dorsal part of the somite. The cells in the dermomyotome undergo division and migration and produce myoblasts. The myoblasts then migrate into the limb bud [4,5], where they proliferate, differentiate, fuse, and ultimately form skeletal muscle. The molecular regulation of embryonic myogenesis has been partially established in mammals, particularly in mice [6]. The main transcription factors regulating the myogenic lineage include Six1/4 transcription factors, the paired-homeobox transcription factors Pax3/7, and the family of myogenic regulatory factors (MRFs), including myogenic factor 5 (Myf5), myogenic differentiation 1 (MyoD), myogenin (MyoG), and myogenic regulatory factor 4 (MRF4) (Figure 1). Six1/4 and Pax3/7 are primary regulators of early lineage specification. Myf5 and MyoD commit cells to the myogenic program, while MyoG and MRF4 jointly determine the expression of terminal differentiation genes required for muscle cell fusion and myotube formation [7]. The MRFs control the formation of skeletal muscle. It has been reported that when members of the MRF family are overexpressed in non-muscle cells, they will activate the myogenic program and inhibit other cell fates and the formation of differentiated muscle [8]. The MRF family has four members, among which MyoD, Myf5, and MRF4 are myogenic determination factors. Without these three, skeletal muscle formation would not occur. As a differentiation factor, MyoG controls the differentiation of myoblasts into skeletal muscle fibers [9,10]. MyoG-knockout homozygous mice have normal myoblasts that can survive during embryonic development but die immediately after birth and show a severe reduction in all skeletal muscles [11,12]. MRFs control entry into myogenic programs, leading to the formation of skeletal muscle. However, there are other important transcription factors that guide cells to undergo myogenesis upstream of them. Six1/4 and Pax3/7 are important upstream regulators [13,14]. The ectopic expression of Six1 leads to the activation of Pax3 and myogenic regulatory genes [15]. Six1/Six4 double-KO mice exhibit severe craniofacial and rib defects, as well as general muscle hypoplasia [16]. Pax3 plays a major role in early embryonic skeletal muscle formation. Knocking out Pax3 in the embryonic phase affects the production of somatic cells, leading to abnormal myotome formation and trunk muscle defects [17]. Most significantly, the muscles in the limbs disappear [18,19,20]. Pax7 dominates in postnatal muscle growth and regeneration. Pax7 is essential for maintaining resting satellite cells and muscle regeneration [21,22]; however, it is dispensable and its function can be replaced by Pax3 during embryonic development [23].

The number of skeletal muscle fibers is determined during embryonic development, and the growth of skeletal muscle after birth mainly depends on the accumulation of muscle-specific proteins and the proliferation of muscle satellite cells. Muscle satellite cells are a type of mononuclear cell located between the basement membrane and muscle fiber membrane that remain quiescent under normal conditions [24,25]. When muscles are damaged or subjected to mechanical loads, they are activated immediately to express the Pax7 gene, and begin to proliferate, differentiate, and fuse, ultimately forming new muscle fibers to supplement the injured site [26,27]. Adult skeletal muscle undergoes changes in size and metabolic activity due to extracellular and intracellular signals [28]. Exercise, nutrient intake, and several growth factors can lead to the accumulation of new proteins and organelles in the cytoplasm, increasing cell volume, which is a process known as hypertrophy. On the contrary, a lack of exercise and many chronic disease states can promote a net loss of proteins, organelles, and cytoplasm, leading to a reduction in cell volume, a condition known as atrophy [29]. Skeletal muscle degeneration also occurs in many muscle diseases, such as sarcopenia [30,31].

Transcription regulatory factors control embryonic muscle generation and postnatal muscle regeneration processes. Myogenesis is also regulated by epigenetic regulation. Epigenetic regulation controls gene expression by chemically modifying histones, DNA, or RNA without altering the underlying gene sequence [32,33]. The establishment and removal of these chemical marks are regulated via a variety of chromatin-modifying enzymes that can alter chromatin dynamics. Chromatin-modifying enzymes are also recruited to muscle gene regulatory regions, where they coordinate transcription regulatory factors to affect gene expression [34]. Myogenesis is accompanied by dynamic changes in global chromosome modification, especially histone modification in myogenic genes [34,35,36]. In this review, we summarize recent advances, addressing the importance of histone modification in skeletal muscle development, regeneration, and disease.

## 2. Histone Modifications in Muscle Development and Regeneration

At present, multiple types of histone modifications have been identified, including acylation (acetylation, crotonylation, lactylation, etc.), methylation, phosphorylation, sumoylation, dopamine acylation, ubiquitination, ADP-ribosylation, glycosylation, and serotonylation [37,38,39,40,41,42]. These modifications regulate biological processes such as cell proliferation, differentiation, and apoptosis by altering chromatin structure and gene expression [43]. During skeletal muscle development and regeneration, histone modifications regulate the expression of key myogenic genes, affecting the differentiation of muscle stem cells (satellite cells), the formation of muscle fibers, and the maintenance of mature muscle cells. Histone methylation and acetylation modifications play a crucial role in regulating the fate determination of muscle cells. Here, we have summarized the latest progress on histone modifications in muscle development and regeneration.

### 2.1. Role of Histone Methylation

Histone methylation is a reversible post-translational modification catalyzed by histone methyltransferases (HMTs) and histone demethylases (HDMs). Typical lysine methylation occurs at sites such as H3K4, H3K9, H3K27, H3K36, H3K79, and H4K20, while arginine methylation predominantly occurs at sites like H3R2, H3R8, H3R17, H3R26, H2AR3, and H4R3 [44]. The process of histone methylation depends on specific enzymes called histone methyltransferases, which can be broadly classified into lysine-specific methylases and arginine-specific methylases. Lysine-specific methyltransferases (KMTs) are further categorized into two groups: those containing an SET domain (e.g., SUV39H1 and the MLL, EZH, SMYD, and PRDM families) and those lacking an SET domain, such as DOT1L [45,46,47]. Histone demethylation (HDM) involves the removal of methyl groups, converting methylated residues back to their unmethylated form, and is facilitated by distinct enzyme systems and regulatory pathways. HDMs are classified into the lysine-specific demethylase 1 (LSD1) family and other demethylases, with the LSD1 family resembling hydroxylases and playing a key role in demethylation [48]. This process is crucial for regulating the chromatin structure and gene expression as the reversible nature of methylation and demethylation activities influences chromatin accessibility and gene expression levels. The interplay between these enzymes ensures the dynamic regulation of histone modifications, maintaining a balance between gene activation and repression in cellular processes.

Myoblast differentiation requires the loss of inhibitory markers and the addition of permissive markers in the promoter regions of muscle genes. Methylation modifications on H3K9, H3K27, and H4K20 are inhibitory markers enriched on heterochromatin or facultative heterochromatin and are negatively correlated with gene expression, whereas those on H3K4, H3K36, and H3K79 are permissive markers enriched on euchromatin and are positively related to gene expression [49]. The synergistic effect of these markers plays a crucial role in regulating the determination of muscle cells’ fates (Table 1).

#### 2.1.1. H3K9 Methylation Modification

The trimethylation of H3K9 is mediated by the methyltransferase Suv39H1 [74,75]. The main function of Suv39H1 is to maintain myoblasts in the proliferative stage. During the proliferation of myoblasts, Suv391H1 is recruited by MyoD to the promoter regions of muscle target genes such as *MyoG*, leading to H3K9me3 and gene suppression [50,51]. This process requires p38γMAPK to phosphorylate MyoD [52]. Suv39h1 also plays a functional role in mediating the muscle-specific genes involved in the process of terminal differentiation [53]. The overexpression of Suv391H1 in C2C12 cell lines represses MyoD-dependent muscle gene expression and myogenic differentiation [52].

G9a is a histone methyltransferase containing the SET domain [76]. During myoblast proliferation, the homologous domain repressor Msk1 interacts with G9a [54], causing G9a to deposit H3K9me2 on *MyoD*, inhibiting its transcriptional activity and myogenic differentiation [55]. However, in vivo experiments found that there were no phenotypic changes in the skeletal muscle of mice after G9a gene knockout [77]. In addition, a basic helix–loop–helix transcription factor, Sharp-1, inhibits myogenesis by interacting directly with G9a. Sharp-1 overexpression in muscle cells increases the G9a-dependent histone H3K9me2 and MyoD methylation [78].

LSD1, which was the first histone demethylase to be discovered, can demethylate mono- or dimethylated lysine residues 4 and 9 in histone H3 (H3K4 and H3K9) [48]. During muscle differentiation, MEF2 recruits histone demethylase LSD1 to the promoter region of muscle-specific genes to remove H3K9me2 and H3K9me3 inhibitory markers, promoting muscle cell differentiation [56]. The inhibition of LSD1 can maintain H3K9me2 inhibitory markers on the *MyoG* and *MCK* promoter regions, inhibiting myoblast differentiation [56]. In addition, the Suv39h1-mediated removal of H3K9 methylation requires JMJD2A (Kdm4a), and JMJD2A can also promote the demethylation of LSD1 [79].

KDM4A is another H3K9 demethylase required for skeletal muscle differentiation [80]. In proliferating myoblasts, KDM4A reduces the enrichment of H3K9me3 at the promoter of *Myf5*. The upregulation of Myf5 then increases the expression of Cyclin D1, thereby promoting the proliferation of myogenic cells. After differentiation, KDM4A demethylates H3K9me3 at the MyoD and MyoG sites, promoting the expression of muscle-specific genes such as *MyoD*, *MyoG*, and *MyHC*, and thereby promoting myogenic differentiation [57].

It is worth mentioning that H3K9 methylation is usually mutually exclusive with H3K4 methylation [81]. Therefore, in order to maintain the demethylation of H3K9, Set7/9 methyltransferase must deposit H3K4me1 on *MyoG* to ensure that H3K9me3 is not reintroduced [64].

#### 2.1.2. H3K27 Methylation Modification

H3K27me3 is present in myoblasts and myotubes and regulates differentiation by inhibiting muscle-specific genes, such as *MyoD* and *MyoG* [82,83]. H3K27me3 is catalyzed by the polycomb repressor complex 2 (PRC2) subunit EZH2. After activation and the lineage commitment of satellite stem cells, H3K27me3 inhibits the differentiation muscle-specific genes, allowing for cell proliferation. During this process, EZH2 is first phosphorylated by p38 and then recruited by YY1, Jarid2, and others to the promoter of the regulatory gene, catalyzing H3K27me3 and leading to gene inactivation [58,59]. During differentiation, the promoter regions of differentiation regulatory genes such as *MyoG* lose H3K27me3 and are activated. This process is mainly mediated by UTX, a member of the KDM6 family [62]. UTX is recruited to the regulatory regions of target genes such as *MyoG* by the homologous box protein Six4, resulting in the removal of H3K27me3 methylation on the target genes. The demethylation activity of UTX requires RNA PolII elongation to spread throughout the genome [62].

Msk1 kinase also plays an important role in the elimination of the muscle-specific gene H3K27me3, mainly working indirectly through the phosphorylation of histone three serine 28 (H3S28) [60]. However, although Msk1 reduces the binding of Ezh2 to the target gene promoter, it increases Ezh1 binding. Ezh1 has weak H3K27me3 activity [61], and in contrast to EZH2, it is required for myogenic differentiation. EZH1 depletion inhibits muscle differentiation and the recruitment of MyoD to the *MyoG* promoter [60,84]. In addition, the presence of Ezh1 is necessary for recruiting RNA Pol II to *MyoG* for transcription [84].

The incorporation of histone variant H3.3 into differentiation-specific genes is necessary for gene activation [85]. The knockout of H3.3 reduces H3K27me3 and promotes the differentiation of myoblasts. However, H3.1 has the opposite effect [86]. In addition, MEF2 can transform H3.1 into H3.3 through histone chaperone HIRA, eliminating the inhibition of gene activation by H3K27me3 [87].

Once the H3K27me3 label is removed, the TrxG complex (Ash2L) is activated by Mef2d and Six1, which deposit trimethylation on H3K4, forming a euchromatic structure that allows for corresponding gene expression [69].

#### 2.1.3. H4K20 Methylation Modification

The deposition of H4K20me2/3 is of great significance in maintaining the quiescent state of skeletal muscle stem cells. The deposition of H4K20me2/3 is mediated by an H4K20 dimethyltransferase, Suv4-20h1, which controls the quiescent state of MuSCs by promoting the formation of facultative heterochromatin (fHC) and depositing H4K20me2 at the MyoD locus. The deletion of Suv4-20h1 reduces fHC and induces the transcriptional activation and repositioning of the MyoD locus, leading to MuSC activation, stem cell exhaustion, and long-term impaired muscle regeneration [63]. Interestingly, the absence of SUV4-20H1 results in a decrease in the expression level of H3K20me2 and a significant decrease in the level of H3K27me3 [63].

#### 2.1.4. H3K4 Methylation Modification

H3K4me1 is one of the recognized permissible biomarkers, catalyzed by the histone methyltransferase Set7, that directly interacts with MyoD to regulate gene expression [64]. Set7 expression is increased during myogenic differentiation. The inhibition of Set7 leads to a decrease in H3K4me1 levels; the loss of *MyoD*, *MyHC*, and *MyoG* expression; and the impairment of skeletal muscle differentiation [64].

In the open chromatin region, H3K4me1 and H3K4me3 are flanked in a bimodal pattern [88]. Trithorax group (TrxG) proteins with an SET domain catalyze H3K4me3 [89]. In satellite stem cells, the transcription factor Pax7 binds to the *Myf5* promoter region, while the arginine methyltransferase CARM1 methylates the amino terminal arginine residue of Pax7. The methylated Pax7 recruits the TrxG complex (Ash2L/MLL2 methyltransferase) to target the *Myf5* promoter region, deposit H3K4me3, and activate gene expression for myoblast proliferation [65,66,67,68]. Ash2L/MLL2 can also be recruited to the *MyoG* promoter region through interacting with phosphorylated MEF2D by p38-α, which leads to the deposition of H3K4me3 and the promotion of myogenic differentiation [69].

The trithorax homolog methyltransferase MLL5 contains SET and PHD domains but lacks intrinsic HMT activity, regulating H3K4 methylation indirectly. MLL5 may regulate H3K4 methylation by affecting the expression of the histone-modifying enzymes LSD1 and SET7/9. MLL5 is induced in quiescent myoblasts and regulates both the cell cycle and differentiation through a hierarchy of chromatin and transcriptional regulators [70].

PARP1 is a chromatin-related enzyme that typically involves the poly ADP-ribosylation of chromatin proteins. PARP1 is reported to affect H3K4me3 in a complex manner. It is reported that active PARP1 suppresses the histone demethylase KDM5B and increases the accumulation of H3K4me3 on multiple gene promoters [90], while inactive PARP1 inhibits methyltransferase MLL1 and decreases H3K4me3 accumulation at the *IL6* promoter [91]. In skeletal cells, PARP1 is downregulated during myogenic differentiation, and its absence enhances the upregulation of MyoD target genes such as *p57*, *myoglobin*, *Mef2C*, and *p21*. Research has found that PARP1 inhibits the recruitment of MyoD by interacting with some MyoD-binding regions, impairing the accumulation of the license marker H3K4me3 at the MyoD binding site and thereby inhibiting the expression of myogenic genes and myogenic differentiation [71].

#### 2.1.5. H3K36 Methylation Modification

H3K36me3 is an active chromatin marker mediated by 2 (Setd2) containing the SET domain [47,72]. The inhibition of Setd2 in C2C12 cells increases the expression of cyclin-dependent kinase inhibitor p21 and reduces the expression of MyHC and MyoG, thereby inhibiting myoblast proliferation and differentiation [72,73].

### 2.2. Role of Histone Acetylation

Histone acetylation is catalyzed by histone acetyltransferases (HATs). These enzymes catalyze the transfer of acetyl groups from acetyl CoA to specific lysine residues on histones, leading to histone acetylation. As acetylation specifically occurs on lysine residues, they are also known as lysine acetyltransferases (KATs) [92,93,94]. The acetylation of histones causes chromatin to enter a looser state, promoting the binding of transcription factors and RNA polymerase to specific regions of chromatin and activating gene expression [95,96]. On the contrary, histone deacetylase (HDAC) removes acetyl groups from histones, resulting in the formation of closed chromatin structures and inhibiting gene expression [96,97]. HATs are divided into three main families: the GNAT (general control non-repressible/GCN5-related N-acetyltransferases) family, including Gcn5/KAT2A, PCAF/KAT2B, Ada, and SGAG; the MYST family, which comprises KAT5 (TIP60), KAT6A (MOZ/MYST3), KAT6B (MORF/MYST4), KAT7 (HBO1/MYST2), KAT8 (MOF/MYST1), SAS2, and SAS3; and the P300/CBP family, which includes p300/KAT3B and CBP/KAT3A [98]. In addition, transcription factor IIIC (a general transcription factor of RNA polymerase III) and CLOCK (an epigenetic regulator of circadian rhythms in skeletal muscle) possess histone acetyltransferase activity [98,99]. A total of 18 HDACs have been identified and are divided into four categories. Class I HDACs include HDACs 1, 2, 3, and 8; while Class II HDACs are further divided into Classes IIa and IIb. Class IIa includes HDACs 4, 5, 7, and 9, while Class IIb includes HDACs 6 and 10. Class III consists of the SIRT1, 2, 3, 4, 5, 6, and 7 members of the Sirtuin family. Class IV is represented by HDAC11. Currently, multiple histone acetylation regulators are recognized for their involvement in the regulation of muscle development and regeneration (Table 2).

The GNAT family primarily consists of general control non-derepressible 5 (GCN5), P300/CREB binding protein-associated factor (PCAF), Ada, and SGAG [98]. They utilize their histone acetyltransferase activity to regulate genes and transcription factors involved in skeletal muscle formation and regeneration. GCN5 disrupts the interaction between the YY1 zinc finger region and DNA by acetylating YY1, maintaining the expression of key structural muscle proteins and preserving muscle integrity [100]. In human myoblasts, PCAF is recruited to the nuclear lamina by lamin A/C to permit HDAC2 acetylation and displacement from MyoD, promoting myogenic differentiation [101].

The MYST family primarily consists of KAT5 (TIP60), KAT6A (MOZ/MYST3), KAT6B (MORF/MYST4), KAT7 (HBO1/MYST2), KAT8 (MOF/MYST1), SAS2, and SAS3. Some members of the MYST family are involved in muscle regeneration processes. For example, Tip60 recruits MyoD to the myogenic gene promoter by interacting with MyoD, enhancing the transcriptional activity of myogenic regulatory genes. Knocking out Tip60 in C2C12 cells inhibits myogenic cell differentiation [102]. In the early stage of C2C12 myoblast regeneration, the expression of HBO1 is upregulated to cope with cardiotoxin-induced muscle injury [116], suggesting that HBO1 may play a role in skeletal muscle regeneration.

The P300/CBP family primarily consists of P300 and cAMP response element-binding protein-binding protein (CBP). A functional compensation mechanism exists between P300 and CBP in skeletal muscle. Studies have shown that the dual knockout of P300 and CBP leads to rapid changes in gene expression patterns related to skeletal muscle function. These changes result in the loss of contractile function in mice and ultimately lead to death within one week. However, while knocking out P300 or CBP alone may partially impair skeletal muscle function, it remains sufficient to maintain normal physiological activity and does not result in a lethal phenotype [117]. P300 can regulate myogenesis in various ways. For example, p300 activates target genes by interacting with the basic helix–loop–helix (bHLH) domain of tissue-specific transcription factors, thereby regulating skeletal muscle cell differentiation [103]. p300 regulates the cell fate determination of myoblasts by acting genetically upstream of *Myf5* and *MyoD* through its histone acetyltransferase (HAT) activity [104]. p300 can be phosphorylated and activated by Akt/protein kinase B (PKB), thereby mediating myoblast differentiation [118].

Multiple HDACs have been reported to play important roles in myogenesis and muscle regeneration. In Class I HDACs (including HDAC1, 2, 3, and 8), HDAC1 inhibits MyoD-dependent transcription via direct binding [105]. The basal sumoylation of histone deacetylase 1 (HDAC1) increases the deacetylation of MyoD in undifferentiated myoblasts, whereas further sumoylation of HDAC1 helps to convert its binding partners from MyoD to the tumor suppressor Rb to induce myogenic differentiation [119]. HDAC3 regulates myoblast differentiation by activating the myotonic dystrophy gene EMD and reducing the histone acetylation marker H4K5ac [106]. HDAC3 also serves as a crotonylation eraser to decrease AKT1 crotonylation, activate AKT1, and promote myogenic differentiation [107]. HDAC8 interacts physically with EZH2, activating the Wnt signaling pathway to regulate skeletal muscle cell differentiation [120]. HDAC8 also functions as a potential feedback regulator of PKD phosphorylation to control myogenic gene expression [121].

Class II deacetylases, such as HDAC4 and 5, can interact with and inhibit the activity of MEF2 family members [122,123]. Further research found that the nuclear export of HDAC4 and 5 was responsible for the disinhibition of MEF2 in differentiated muscle cells [124]. HDAC4 promotes skeletal satellite cell proliferation and differentiation by inhibiting the transcription of the cell cycle inhibitor Cdkn1a and repressing the expression of the Sharp1 gene, respectively [108]. HDAC4 also mediates muscle regeneration in vivo through soluble factors [109], and HDAC4 regulates the proliferation and differentiation of chicken skeletal muscle satellite cells [125]. HDAC9 regulates skeletal muscle cell differentiation through a negative feedback loop. HDAC9 is a direct transcriptional target of MEF2 in vitro and in vivo, and HDAC9 can bind to the MEF2 protein and inhibit its transcriptional activity [110].

Class III HDACs mainly include the SIRT protein family, which consists of SIRT1-7. SIRT1 and SIRT3 are highly expressed in slow muscle fibers. SIRT1 enhances muscle fatigue resistance during the muscle injury repair process [111]. SIRT3 has three different protein subtypes, among which the overexpression of SIRT3M3 activates AMPK and PPARδ, promoting slow muscle fiber generation [113]. SIRT2 plays an important role in the process of repair following skeletal muscle injury. It actively regulates skeletal muscle cell regeneration by upregulating myogenic regulatory factors (Myf5, MyoD, and Myog) and cell cycle regulatory factors (Cyclin D1, CDK2), and downregulating the muscle weakness gene atrogin1 [112,126]. SIRT6 is crucial in regulating the transition of the muscle fiber structure to the oxidative type as it downregulates Sox6 by increasing the transcription of *CREB*, a key repressor of slow-fiber-specific genes [127].

HDAC11 shows significant activity during the differentiation of C2C12 myoblasts, and its ectopic expression completely inhibits the differentiation of myoblasts. Further research has found that HDAC11 inhibits myoblast differentiation by downregulating MyoD-dependent transcription [114]. HDAC11 also plays an important role in skeletal muscle regeneration. HDAC11 deficiency promotes regeneration after muscle injury. The inhibition of HDAC11 activity upregulates the expression of IL-10 (a known cytokine that promotes myoblast differentiation), thereby promoting muscle regeneration [115]. HDAC11 also promotes the proliferation of bovine skeletal muscle satellite cells by activating the Notch signaling pathway [128].

### 2.3. Role of Other Histone Modifications

Aside from histone methylation and acetylation, there are few reports on the effects of histone modifications on skeletal muscle development. However, recent research has discovered a new histone modification, histone lactylation, which is involved in the regulation of skeletal muscle development. Histone lactylation is a novel epigenetic code which was first reported in 2019 by Zhang et al. [129]. Histone lactylation refers to the addition of a lactyl (La) group to the lysine amino acid residue at the tail of histones. Multiple histone lactate sites have been identified, including H3K4 and H3K18. Histone lactylation links metabolism and gene regulation, playing a crucial role in various biological processes, particularly through its specific functions in a variety of diseases [130]. The specific mechanisms of histone lactylation in skeletal muscle have not been fully elucidated. However, it has been reported that lactate can promote myogenic differentiation [131,132,133]. A further study showed that many promoters and enhancers gain H3K18la during the conversion from myoblasts to myotubes, and genes with an H3K18la promoter peak in myotubes were, on average, slightly upregulated in myoblasts treated with 10 mM lactate [134]. These results suggest that lactate could promote myogenic differentiation by increasing the histone lactylation of promoter genes. Mechanistically, lactate preferentially increases the lactylation of H3K9, enhancing the transcription of *Neu2*, which is believed to promote myoblast differentiation [135]. Histone lactylation also plays an important role in ischemia-induced muscle regeneration by affecting the function of macrophages [136].

## 3. Histone Modifications in Skeletal Muscle Atrophy

Histone modifications exhibit dynamic changes in various forms of muscle atrophy. As age increases, the overall acetylation levels of histones H3K9 and H3K27 and the trimethylation level of H3K9 decrease in the gastrocnemius muscle in rats, which may be related to age-related skeletal muscle atrophy [137]. Increased pan acetylation of histone H3 is observed in skeletal muscle atrophy induced by denervation [138]. Similarly, in muscle atrophy caused by hind limb plaster fixation, histone acetylation increases [139]. Different types of muscles also exhibit different histone modifications. For example, a study found differences in histone modifications between the plantar (fast) and soleus (slow) muscles of adult rats through chromatin immunoprecipitation and DNA sequencing. The authors found that the activation of fast genes in the plantaris is associated with enhanced H3K4me3 and H3 acetylation, while the transcription of slow genes in the soleus is independent of H3K4me3 and H3 acetylation [138]. These studies suggest that histone methylation and acetylation modifications may be involved in the regulation of various types of muscle atrophy.

The role of histone methylation and acetylation modifications in skeletal muscle atrophy have been summarized in detail in the literature [140]. In this section, we focus on summarizing the important regulatory roles of histone methylation and acetylation in different muscle atrophy models. Histone lysine methyltransferases are divided into two categories: histones with SET domains (including the SUV39, MLL, EZH, SMYD, and PRDM families) and the non-SET-domain protein DOT1L. In Duchenne muscular dystrophy, high concentrations of inflammatory mediators such as TNF-α activate the NF-κB signaling pathway, promoting the recruitment of EZH2 and Dnmt3b to the *Notch-1* gene promoter region. This results in the epigenetic silencing of *Notch-1*, impairing the regenerative ability of SCs and promoting muscle degeneration [141,142]. In denervation-induced skeletal muscle atrophy, MLL1 expression is first increased and then decreased, which suggests that it may regulate this process [143]. In primary skeletal muscle cells and C2C12 myogenic cells, the SMYD3 family member SMYD3 recruits BRD4 to its regulatory region through protein interactions. This favors the chromatin engagement of the pause–release factor p-TEFb (positive transcription elongation factor) and the elongation of Ser2-phosphorylated RNA polymerase II, ultimately increasing the transcription of *myostatin* and *c-Met* and thereby exacerbating glucocorticoid-induced muscular atrophy [144]. Furthermore, in a mouse model of dexamethasone-induced skeletal muscle atrophy, SMYD3 depletion prevented muscle loss and a decrease in fiber size [144]. Histone demethylases are also involved in the regulation of skeletal muscle fiber atrophy. The depletion of LSD1 in skeletal muscle fibers reduces the nuclear retention of the autophagic transcription factor Foxk1 by inhibiting the Akt-mTORC1 axis, thereby amplifying glucocorticoid-induced fast muscle fiber atrophy [145].

The role of histone acetylation regulators in skeletal muscle atrophy has been widely studied. The P300/CBP family P300/CBP plays an important role in various types of muscle atrophy models, mainly by regulating FOXO family transcription factors [145]. P300/CBP can inhibit FOXO expression at the transcriptional and post-translational levels through histone acetylation [146] and non-histone acetylation, respectively [147]. FOXO then targets the promoter of *MAFbx*, causing the rapid transcription of *MAFbx* and skeletal muscle atrophy [148]. In L6 muscle cells treated with dexamethasone (a commonly used in vitro model of muscle atrophy), the downregulation of p300 can reduce the acetylation of the transcription factors C/EBP, FOXO, and p65; decrease the expression of the ubiquitin ligase MuRF1; and reduce dexamethasone-induced muscular atrophy [149]. Similarly, enhancing p300 HAT activity in vivo is sufficient to block FOXO activation in response to skeletal muscle disuse and in C2C12 cells in response to nutrient deprivation or dexamethasone treatment [146]. In disease- or cancer-induced muscle atrophy models, CBP/p300 is typically phosphorylated, and phosphorylated CBP/p300 enhances its acetyltransferase activity, leading to morphological and molecular changes associated with atrophy [150,151,152,153].

In addition, the GCN5 family member PCAF can be recruited to the nuclear envelope by lamin A/C. The expression of lamin A/C mutation inhibits the translocation of PCAF to the nuclear envelope, thereby impairing the differentiation of myoblasts and resulting in Emery–Dreifuss muscular dystrophy myoblasts [101].

Multiple HDACs mediate skeletal muscle atrophy. Like P300, HDAC1 can increase the expression of the muscle atrophy gene atrogin-1 by activating the FOXO signaling pathway, leading to skeletal muscle atrophy [154]. HDAC1 is necessary for muscle atrophy related to skeletal muscle disuse [154]. A reduction in the level of HDAC1 or the inhibition of its activity prevents muscle atrophy after nutrient deprivation [154]. Additionally, HDAC1 specifically inhibits *miR-206* in dystrophic MuSCs; therefore, it is associated with several pathogenic features of DMD [155]. HDAC2 expression is increased in the muscles of muscle dystrophin-deficient MDX mice. The downregulation of HDAC2 expression leads to improved mdx MuSC myogenesis in vitro, as well as improved functional and morphological parameters in vivo [156]. The expression of HDAC8 is increased in DMD patients and a zebrafish DMD model. HDAC8 inhibition through the administration of PCI-34051 is able to rescue the DMD phenotype [157].

Class II HDACs may play a role in muscular dystrophy as SIK1, a Class II HDAC kinase, can improve the dystrophic phenotype in mice expressing a dominant-negative CREB transgene [158]. Class II HDACs also play a role in neurogenic muscle atrophy. HDAC4 is specifically upregulated in animal models of disease and human diseases resulting from neurogenic muscle atrophy [159,160]. In a mouse model of Amyotrophic Lateral Sclerosis (ALS), the deletion of HDAC4 in skeletal muscle worsens the pathological features of the disease, exacerbating skeletal muscle loss and denervation [160]. Another study reported that HDAC4 and HDAC5 regulated the transcription factor MyoG to execute muscle atrophy after the loss of innervation by motor neurons [161]. Additionally, in a hypoxia-induced muscle atrophy cell model, the expression of HDAC9 significantly increases, inhibiting intracellular autophagy levels and regulating muscle fiber atrophy [162]. The pharmacological inhibition of HDAC6 improves muscle phenotypes in dystrophin-deficient mice by downregulating TGF-beta via Smad3 acetylation [163].

SIRTs are closely related to skeletal muscle atrophy. In both C2C12 cell and rat models of hyperglycemia-induced skeletal muscle atrophy, the expression of multiple SIRTs was found to change significantly, suggesting that SIRTs may play an important role in this process [164]. Further research found that lespedeza bicolor extract, which has an antidiabetic activity, can improve skeletal muscle atrophy by activating SIRT1, SIRT3, SIRT4, and PGC1α [165]. SIRT1 can also inhibit type I fiber atrophy during intermittent fasting by deacetylating and suppressing the transcriptional activity of FoxO1 and FoxO3 [166]. In addition, activating SIRT1 in muscle cells can prevent a decrease in the expression of type I myosin heavy chain genes and glucose-induced myotube atrophy [167]. In a mouse model of Duchenne muscular dystrophy, the overexpression of SIRT1 in muscles increases slow muscle fibers and improves the muscle atrophy phenotype [168]. In a mouse model of dexamethasone-induced muscle atrophy, the inhibition of SIRT2 significantly reduces muscle mass and endurance capacity. On the contrary, the overexpression of SIRT2 alleviates dexamethasone-induced myotube atrophy in vitro [126]. SIRT3 is highly expressed in slow muscle fibers, and the overexpression of SIRT3 increases levels of FoxO1 transcription factor and its downstream muscle atrophy gene MuRF-1, leading to muscle atrophy [113].

## 4. Histone Modifications in Skeletal Muscle Hypertrophy

Skeletal muscle hypertrophy, which is characterized by an increase in muscle fiber diameter and strength output [169], requires the activation of satellite cells [170]. Skeletal muscle mass is positively regulated by the insulin-like growth factor-1 (IGF-1)/Akt and β-adrenergic pathways and is negatively regulated by myostatin, NF-κβ, and glucocorticoid signaling [171]. Histone modifications also play a role in skeletal muscle hypertrophy. Currently, there is little research on the regulation of skeletal muscle hypertrophy by histone modifications, with most studies focusing on the effects of histone acetylation modifications.

Resistance exercise training induces skeletal muscle hypertrophy, thereby increasing muscle strength. It is reported that H3K36 acetylation significantly increases following exercise [172,173]. In addition, the overexpression of HDAC5 in mouse skeletal muscle is sufficient to attenuate adaptations to exercise training [174]. These studies suggest that histone acetylation may play a role in muscle hypertrophy in response to exercise. Under normoxic and hypoxic conditions, pulmonary inflammation can completely suppress Wistar rat soleus muscle hypertrophy in response to surgical overload. The effect of pulmonary inflammation may be due to changes in histone acetylation signaling. The level of histone H3 acetylation is significantly increased under hypoxic conditions and the inhibition of hypertrophy in the presence of inflammation is totally reversed by the administration of the inhibitor I-BET151, a drug that specifically targets epigenetic signaling [175], suggesting that histone acetylation may also play a role in the overload-induced hypertrophy of skeletal muscle.

However, the mechanism by which histone acetylation regulates skeletal muscle hypertrophy is not clear. Only a few studies have reported the regulatory effect of histone deacetylase on skeletal muscle hypertrophy. For example, HDAC4 is the best characterized repressor of the pro-hypertrophic transcription factor MEF2. The HDAC4 nuclear export leads to MEF2 activation, and constitutively active MEF2 is able to induce adult muscle myofiber hypertrophy [176]. In addition, *miR-206* represses myogenic cell hypertrophy by inhibiting HDAC4 [177]. SIRT1 may play a crucial role in the overload-induced hypertrophy of skeletal muscle [178]. SIRT1 could enhance protein synthesis through IGF-1–AKT signaling and decrease protein degradation through FOXO1 inactivation [178]. In addition, SIRT1 can activate Pax7-dependent satellite cells and regenerate skeletal muscle, an early event in the development of muscle hypertrophy [178].

## 5. Histone Modifications and Human Skeletal Muscle Diseases

### 5.1. Histone Modifications in DMD

Duchenne muscular dystrophy (DMD) is an X-chromosome-linked disease caused by loss-of-function mutations in the human dystrophin gene, leading to severe and progressive muscle wasting [179,180]. Recent research has highlighted the role of epigenetic modifications, particularly histone modifications, in the pathogenesis of DMD. In the context of DMD, histone modifications in skeletal muscle cells typically undergo changes. For example, a study by Brenman et al. reported that muscle samples from DMD patients and animal models were enriched in specific histone H3 modifications, including Ser-10 phosphorylation, the acetylation of Lys 9 and 14, and Lys 79 methylation. These modifications were associated with genes involved in proliferation and inflammation, indicating a regulatory effect of histone modifications on gene expression in dystrophic muscles [181]. In addition, the dysregulated inflammatory response observed in DMD is associated with epigenetic changes involving histone acetylation. The increase in HDAC activity helps to inhibit muscle regeneration factors and promotes chronic inflammation, fibrosis, and fat production in dystrophic muscles [182], indicating that HDACs play an important role in DMD. In fact, multiple HDACs have been reported to be involved in the pathogenesis of DMD (see review [183]). Specifically, the expression of HDAC8 of the Class I HDACs; HDAC4 and 5 of Class II; and SIRT2, 3, 4, and 5 of Class III HDACs was upregulated in the skeletal muscles of DMD patients [157,181,184,185]. Further research using DMD animal or cell models has revealed the involvement of multiple HDACs in the pathogenesis of DMD (see Section 3, “Histone modifications in skeletal muscle atrophy”).

Given the role of HDACs in DMD pathology, HDAC inhibitors (HDACis) have been investigated as potential therapeutic agents. HDACis work by inhibiting the deacetylation of histones, leading to a more relaxed chromatin structure and increased gene expression. This can enhance muscle regeneration and reduce fibrosis in dystrophic muscles. Givinostat (Duvyzat) is an HDACi that has shown promise in clinical trials for DMD [186]. In a phase III study, patients treated with givinostat exhibited less decline in muscle function compared to those receiving a placebo. The FDA approved givinostat in March 2024 for the treatment of DMD in individuals aged six years and older.

### 5.2. Histone Modifications in FSHD

Facioscapulohumeral muscular dystrophy (FSHD) is one of the most common muscular dystrophies in adults. It is characterized by progressive weakness and the loss of skeletal muscle [187]. Unlike many other dystrophies that are caused by mutations resulting in the loss of a protein product, FSHD is associated with the epigenetic dysregulation of the chromosome 4q35 D4Z4 macrosatellite. The ultimate consequence is the ectopic expression of the double homeobox 4 (*DUX4*) gene in skeletal muscle, which is normally suppressed in healthy individuals [188]. Histone modifications are closely related to FSHD—the chromatin state at the *D4Z4* locus is finely regulated by histone modifications. In normal muscles, the chromatin in the *D4Z4* repeat sequence is in a silent state and is labeled with inhibitory histone modifications such as H3K9me3 and H3K27me3. In FSHD muscles, the levels of these inhibitory markers are significantly reduced. Meanwhile, there is an increase in active histone modifications, like H3Ac and H3K4me3, in the *D4Z4* region [189,190,191]. The loss of inhibitory epigenetic modifications and the increase in active histone modifications are directly related to the activation of the *DUX4* gene. These changes in histone methylation and acetylation modifications in FSHD suggest the involvement of specific histone-modifying enzymes. The histone methyltransferases SUV39H1 and EZH2, which catalyze H3K9me3 and H3K27me3, respectively, may be involved in the pathogenesis of FSHD [192]. It is reported that the loss of SUV39H1-dependent H3K9me3 at *D4Z4* in FSHD results in the abolishment of HP1γ/cohesin binding in myoblasts, which has adverse effects on chromatin organization and leads to muscular dystrophy [189]. In addition, histone acetyltransferases p300 and CBP interact with DUX4 and are recruited to the target genes of DUX4. Interference with this interaction can inhibit DUX4 transcriptional activation [193,194] and is beneficial for the treatment of FSHD.

### 5.3. Histone Modifications in Sarcopenia

Sarcopenia is a degenerative musculoskeletal disease related to aging and the progressive loss of skeletal muscle strength, mass, and function [195]. Histone methylation and acetylation undergo significant changes with aging, and many studies have reported the important role of histone-modifying enzymes in the aging process [196], indicating that histone methylation and acetylation are closely related to aging. A recent study investigated the age-related overall changes in histone modifications in rat gastrocnemius muscle. They found that overall histone H3 methylation and acetylation decreased with age, and the decrease in histone acetylation may have been related to age-related muscle atrophy in the gastrocnemius muscle of rats [137]. These studies suggest that histone methylation and acetylation may play important roles in the pathogenesis of sarcopenia, although the specific functions and potential molecular mechanisms of specific histone-modifying enzymes in sarcopenia are currently poorly understood. Specifically, HDACs regulate metabolic flexibility, which is the ability of muscles to adapt to fuel utilization based on the activity and nutrient availability in skeletal muscle. Impaired metabolic flexibility is associated with sarcopenia. For instance, the Class III HDAC SIRT3 deacetylates proteins involved in mitochondrial function, thereby affecting muscle metabolism [197]. Therefore, HDACs may participate in sarcopenia by regulating muscle metabolism, and serve as therapeutic targets for sarcopenia.

### 5.4. Histone Modifications in Other Skeletal-Muscle-Related Diseases

Cachexia is a skeletal-muscle-related disease and multifactorial syndrome characterized by weight loss, muscle atrophy, and metabolic disorders, resulting in functional impairment and reduced quality of life [198]. These underlying illnesses include cancer, kidney disease, neurological disease, heart failure, chronic obstructive pulmonary disease, and AIDS [199]. Its mechanism involves systemic inflammation and an energy metabolism imbalance. Recent studies have shown that histone modifications may participate in the occurrence of cachexia by regulating metabolic reprogramming and inflammatory responses. For example, the histone deacetylase SIRT6 ameliorates cachexia-associated adipose wasting in cancer by inhibiting TNFR2 signaling in mice. Its functional defects may lead to lipid accumulation and metabolic abnormalities [200], thereby exacerbating energy consumption in cachexia. HDAC3 binds to the rhythm molecule RORA to form an inhibitory complex, which binds directly to the *PD-L1* (CD274) promoter region and inhibits its transcriptional expression by deacetylating histone H3K9. In melanoma models, the abnormal activation of HDAC3 can cause RORA to dissociate from the inhibitory complex, thereby releasing the inhibition of *PD-L1* and promoting immune escape [201]. This may indirectly affect the chronic inflammatory states associated with cachexia. At present, direct research on histone modifications in cachexia remains limited; however, the potential mechanisms of these modifications can be inferred through their roles in metabolic reprogramming and inflammation regulation. In the future, it is necessary to combine single-cell multi-omics technologies (such as scATAC-seq combined with scRNA-seq) to analyze tissue-specific epigenetic regulatory networks and develop precise therapies targeting histone-modifying enzymes to improve the prognosis of cachexia.

## 6. Challenges and Future Perspectives

Histone modifications, especially histone methylation and acetylation modifications, play important roles in skeletal muscle development and regeneration. In addition, histone modifications are involved in the occurrence and development of various skeletal muscle diseases. Histone methylation and acetylation play important roles in pathological processes such as muscle atrophy, hypertrophy, and metabolic disorders. Histone demethylase and deacetylase inhibitors show potential in the treatment of muscle atrophy and other muscle diseases [202]. For example, an HDACi named givinostat (Duvyzat) has shown promise in clinical trials for DMD [186]. Although significant progress has been made in the study of histone modifications in skeletal muscle development and disease, some unresolved issues and challenges remain, including the following:

i. The diversity and complexity of histone modifications make it difficult to fully reveal their specific functions in different biological contexts. For example, the specific mechanisms of some newly discovered histone modifications, such as histone lactylation [134], have not been fully elucidated in skeletal muscle. The application of new technologies will result in breakthroughs in this field. The further development of high-throughput sequencing technology will help to reveal the complex regulatory network of histone modifications, especially dynamic changes at different developmental stages and pathological states. The application of CRISPR/Cas9 gene editing technology can precisely manipulate histone modification-related genes, aiding in the study of their specific functions in determining the fate of muscle cells.
ii. Histone modifications may interact with other epigenetic mechanisms [203,204]. Several histone modifications were found to be involved in regulating miRNA expression in cancer or during its development [205]. In turn, some miRNAs can regulate the expression and enzymatic activity of histone-modifying enzymes in cancer [206]. MiRNA and histone modifications jointly regulate the development of and adaptive changes in skeletal muscle [203]. Histone modifications can also interact with DNA methylation. For example, histone modifications and DNA methylation act cooperatively in regulating symbiosis genes in the sea anemone Aiptasia [207]. However, existing research has mostly focused on a single type of modification and has overlooked the synergistic effects of different modifications, which may lead to a one-sided understanding of muscle development and disease mechanisms. Therefore, understanding the interaction between histone modifications and other epigenetic modifications such as ncRNA will be beneficial for a comprehensive understanding of the regulatory network of histone modifications.
iii. There are multiple technological methods currently available for studying the function of histone modifications. For example, ChIP-seq and CUT&Tag can be used to locate the genomic regions of histone modifications. Mass spectrometry can accurately identify the types, sites, and combination patterns of histone modifications (acetylation, methylation, phosphorylation, etc.), making it particularly suitable for the discovery of unknown modifications. Single cell-ChIP-seq can be used to study histone modification heterogeneity at a single-cell resolution. Although the application of high-throughput sequencing technology provides a powerful tool for histone modification research, challenges remain in data analysis and interpretation. In complex biological systems in particular, further exploration is needed to accurately identify and validate the functional sites of histone modifications and their specific roles in gene regulatory networks.
iv. Sex hormones are crucial for muscle physiological function and seem to be the most probable candidates in the regulation of key molecular pathways of skeletal muscle, with an impact on its mass and functionality [208,209]. Differences in the muscle transcriptome between males and females are largely related to testosterone and estradiol [210]. Testosterone is known to promote muscle protein synthesis and muscle regeneration through its androgen receptor [211]. However, it is currently unclear whether the regulation of the skeletal muscle’s physiological function by sex hormones is mediated by histone modifications. Therefore, an in-depth analysis of the impact of hormone differences on muscle histone modification can not only reveal the molecular mechanisms of differences in muscle development and function between sexes but also provide a valuable theoretical basis for sex-specific intervention strategies for the prevention and treatment of muscle-related diseases.
v. Epigenetic drugs have shown broad prospects in the treatment of skeletal muscle diseases. For example, selective inhibitors targeting HDAC6, such as Tubastatin A, have been shown to stabilize microtubule networks, restore autophagy function, and improve muscle function in DMD mouse models [163]. In spinal muscular atrophy, the combination of the HDAC inhibitor LBH589 and antisense oligonucleotides (such as Nusirersen) significantly enhances the splicing of the SMN2 gene and the expression of the SMN protein [212], suggesting that combination therapy may improve therapeutic efficacy. Nevertheless, existing research has mostly been based on animal models, and determining how to effectively translate these findings into treatment strategies for human diseases remains an important research direction.

In summary, histone modifications play a crucial role in skeletal muscle development and regeneration, as well as muscle diseases. The development of new tools and technologies is conducive to discovering new types of histone modifications. In-depth research on the interactions between different modifications will help to further elucidate the delicate regulatory network of histone modifications, and will ultimately enable the clinical use of various histone-modifying enzyme inhibitors.

## Figures and Tables

**Table 1:** Functional histone methylation modifications and their regulators in skeletal muscle development and regeneration.

Histone Methylation Properties	Histone Methylation Types	Regulator	Function	Mechanism	Ref.
Inhibitory markers	H3K9 methylation	Suv39H1	Maintain myoblasts in proliferative stage	Interact with phosphorylated MyoD; deposit H3K9me2	[50,51,52]
			Inhibit myogenic differentiation	Interact with MyoD; deposit H3K9me3	[52,53]
		G9a	Inhibit myogenic differentiation	Interact with Msk1; deposit H3K9me2	[54,55]
		LSD1	Promote myogenic differentiation	Interact with MEF2; remove H3K9me2 and H3K9me3	[56]
		KDM4A	Promote myogenic differentiation and muscle regeneration	Remove H3K9me3	[57]
	H3K27 methylation	EZH2	Promote myoblast proliferation and inhibit differentiation	Interact with YY1; deposit H3K27me3	[58,59]
		EZH1	Promote myogenic differentiation	Interact with Pol II Complex; deposit H3K4me3	[57]
		Msk1	Promote myogenic differentiation	Phosphorylize H3S28; remove H3K27me3	[60,61]
		UTX	Promote myogenic differentiation	Interact with Six4; remove H3K27me3	[62]
	H4K20 methylation	Suv4-20h1	Maintain quiescent state of skeletal muscle stem cells	Promote fHC formation; deposit H4K20me2	[63]
Permissive markers	H3K4 methylation	Set7	Promote myogenic differentiation	Interact with MyoD; deposit H3K4me1	[64]
		TrxG	Promote myoblast proliferation	Interact with methylated Pax7; deposit H3K4me3	[65,66,67,68]
			Promote myogenic differentiation	Interact with phosphorylated MEF2D	[69]
		MLL5	Promote myoblast proliferation and differentiation	Regulate LSD1 and SET7/9; deposit H3K4me2/3	[70]
		PARP1	Inhibit myogenic differentiation	Interact with MyoD binding regions; remove H3K4me3	[71]
	H3K36 methylation	Setd2	Promote myoblast proliferation and differentiation	Inhibit p21, MyoG, and MyHC	[72,73]

**Table 2:** Functional histone acetylation regulators in skeletal muscle development and regeneration.

Histone Acetylation Properties	Histone Acetylation Types	Regulator	Function	Mechanism	Ref.
HATs	GNAT family	GCN5	Preserve muscle integrity	Interact with and acetylate YY1	[100]
		PCAF	Promote myogenic differentiation	Interact with lamin A/C and acetylate HDAC2	[101]
	MYST family	Tip60	Promote myogenic differentiation	Interact with MyoD	[102]
	P300/CBP family	P300	Promote myogenic differentiation	Bind to bHLH or act upstream of Myf5 and MyoD	[103,104]
HDACs	Class I	HDAC1	Inhibit myogenic differentiation	Interact with MyoD	[105]
		HDAC3	Promote myogenic differentiation	Activate EMD and reduce H4K5ac	[106]
		HDAC8	Promote myogenic differentiation	Interact with EZH2	[107]
	Class II	HDAC4	Promote myoblast proliferation, differentiation, and regeneration	Inhibit Cdkn1a and Sharp1	[108,109]
		HDAC9	Inhibit myogenic differentiation	Interact with MEF2	[110]
	Class III	SIRT1	Improve muscle fatigue resistance after repair from muscle injury	Interact with p53	[111]
		SIRT2	Promote muscle regeneration	Enhance MRFs and CDKs, and inhibit atrogin1	[112]
		SIRT3	Promote formation of oxidative muscle fiber	Activate AMPK and PPARδ	[113]
	Class IV	HDAC11	Inhibit myogenic differentiation and muscle regeneration	Inhibit MyoD activity	[114,115]

## Data Availability

Not applicable.

## References

[1] Yin L., Li N., Jia W., Wang N., Liang M., Yang X., Du G. (2021). Skeletal muscle atrophy: From mechanisms to treatments. Pharmacol. Res..

[2] Buckingham M., Bajard L., Chang T., Daubas P., Hadchouel J., Meilhac S., Montarras D., Rocancourt D., Relaix F. (2003). The formation of skeletal muscle: From somite to limb. J. Anat..

[3] Comai G., Tajbakhsh S. (2014). Molecular and cellular regulation of skeletal myogenesis. Curr. Top. Dev. Biol..

[4] Burke A.C., Nowicki J.L. (2003). A new view of patterning domains in the vertebrate mesoderm. Dev. Cell.

[5] Shearman R.M., Burke A.C. (2009). The lateral somitic frontier in ontogeny and phylogeny. J. Exp. Zool. B Mol. Dev. Evol..

[6] Buckingham M. (2001). Skeletal muscle formation in vertebrates. Curr. Opin. Genet. Dev..

[7] Bentzinger C.F., Wang Y.X., Rudnicki M.A. (2012). Building muscle: Molecular regulation of myogenesis. Cold Spring Harb. Perspect. Biol..

[8] Weintraub H., Davis R., Tapscott S., Thayer M., Krause M., Benezra R., Blackwell T.K., Turner D., Rupp R., Hollenberg S. (1991). The myoD gene family: Nodal point during specification of the muscle cell lineage. Science.

[9] Moncaut N., Rigby P.W., Carvajal J.J. (2013). Dial M(RF) for myogenesis. FEBS J..

[10] Fong A.P., Tapscott S.J. (2013). Skeletal muscle programming and re-programming. Curr. Opin. Genet. Dev..

[11] Hasty P., Bradley A., Morris J.H., Edmondson D.G., Venuti J.M., Olson E.N., Klein W.H. (1993). Muscle deficiency and neonatal death in mice with a targeted mutation in the myogenin gene. Nature.

[12] Nabeshima Y., Hanaoka K., Hayasaka M., Esumi E., Li S., Nonaka I., Nabeshima Y. (1993). Myogenin gene disruption results in perinatal lethality because of severe muscle defect. Nature.

[13] Buckingham M., Relaix F. (2007). The role of Pax genes in the development of tissues and organs: Pax3 and Pax7 regulate muscle progenitor cell functions. Annu. Rev. Cell Dev. Biol..

[14] Buckingham M., Rigby P.W. (2014). Gene regulatory networks and transcriptional mechanisms that control myogenesis. Dev. Cell.

[15] Heanue T.A., Reshef R., Davis R.J., Mardon G., Oliver G., Tomarev S., Lassar A.B., Tabin C.J. (1999). Synergistic regulation of vertebrate muscle development by Dach2, Eya2, and Six1, homologs of genes required for Drosophila eye formation. Genes Dev..

[16] Grifone R., Demignon J., Houbron C., Souil E., Niro C., Seller M.J., Hamard G., Maire P. (2005). Six1 and Six4 homeoproteins are required for Pax3 and Mrf expression during myogenesis in the mouse embryo. Development.

[17] Tremblay P., Dietrich S., Mericskay M., Schubert F.R., Li Z., Paulin D. (1998). A crucial role for Pax3 in the development of the hypaxial musculature and the long-range migration of muscle precursors. Dev. Biol..

[18] Lewis S.E. (1978). Developmental analysis of lethal effects of homozygosity for the c25H deletion in the mouse. Dev. Biol..

[19] Goulding M., Lumsden A., Paquette A.J. (1994). Regulation of Pax-3 expression in the dermomyotome and its role in muscle development. Development.

[20] Bober E., Franz T., Arnold H.H., Gruss P., Tremblay P. (1994). Pax-3 is required for the development of limb muscles: A possible role for the migration of dermomyotomal muscle progenitor cells. Development.

[21] Gunther S., Kim J., Kostin S., Lepper C., Fan C.M., Braun T. (2013). Myf5-positive satellite cells contribute to Pax7-dependent long-term maintenance of adult muscle stem cells. Cell Stem Cell.

[22] von Maltzahn J., Jones A.E., Parks R.J., Rudnicki M.A. (2013). Pax7 is critical for the normal function of satellite cells in adult skeletal muscle. Proc. Natl. Acad. Sci. USA.

[23] Relaix F., Rocancourt D., Mansouri A., Buckingham M. (2005). A Pax3/Pax7-dependent population of skeletal muscle progenitor cells. Nature.

[24] Relaix F., Zammit P.S. (2012). Satellite cells are essential for skeletal muscle regeneration: The cell on the edge returns centre stage. Development.

[25] Persson P.B. (2015). Skeletal muscle satellite cells as myogenic progenitors for muscle homoeostasis, growth, regeneration and repair. Acta Physiol..

[26] Dumont N.A., Bentzinger C.F., Sincennes M.C., Rudnicki M.A. (2015). Satellite Cells and Skeletal Muscle Regeneration. Compr. Physiol..

[27] Sambasivan R., Yao R., Kissenpfennig A., Van Wittenberghe L., Paldi A., Gayraud-Morel B., Guenou H., Malissen B., Tajbakhsh S., Galy A. (2011). Pax7-expressing satellite cells are indispensable for adult skeletal muscle regeneration. Development.

[28] Sandri M. (2008). Signaling in muscle atrophy and hypertrophy. Physiology.

[29] Sartori R., Romanello V., Sandri M. (2021). Mechanisms of muscle atrophy and hypertrophy: Implications in health and disease. Nat. Commun..

[30] Damluji A.A., Alfaraidhy M., AlHajri N., Rohant N.N., Kumar M., Al Malouf C., Bahrainy S., Ji Kwak M., Batchelor W.B., Forman D.E. (2023). Sarcopenia and Cardiovascular Diseases. Circulation.

[31] Sayer A.A., Cruz-Jentoft A. (2022). Sarcopenia definition, diagnosis and treatment: Consensus is growing. Age Ageing.

[32] Goldberg A.D., Allis C.D., Bernstein E. (2007). Epigenetics: A landscape takes shape. Cell.

[33] Fitz-James M.H., Cavalli G. (2022). Molecular mechanisms of transgenerational epigenetic inheritance. Nat. Rev. Genet..

[34] Jin W., Peng J., Jiang S. (2016). The epigenetic regulation of embryonic myogenesis and adult muscle regeneration by histone methylation modification. Biochem. Biophys. Rep..

[35] Barreiro E., Tajbakhsh S. (2017). Epigenetic regulation of muscle development. J. Muscle Res. Cell Motil..

[36] Bharathy N., Ling B.M., Taneja R. (2013). Epigenetic regulation of skeletal muscle development and differentiation. Subcell. Biochem..

[37] Nitsch S., Zorro Shahidian L., Schneider R. (2021). Histone acylations and chromatin dynamics: Concepts, challenges, and links to metabolism. EMBO Rep..

[38] Hagihara H., Shoji H., Otabi H., Toyoda A., Katoh K., Namihira M., Miyakawa T. (2021). Protein lactylation induced by neural excitation. Cell Rep..

[39] Lepack A.E., Werner C.T., Stewart A.F., Fulton S.L., Zhong P., Farrelly L.A., Smith A.C.W., Ramakrishnan A., Lyu Y., Bastle R.M. (2020). Dopaminylation of histone H3 in ventral tegmental area regulates cocaine seeking. Science.

[40] Farrelly L.A., Thompson R.E., Zhao S., Lepack A.E., Lyu Y., Bhanu N.V., Zhang B., Loh Y.E., Ramakrishnan A., Vadodaria K.C. (2019). Histone serotonylation is a permissive modification that enhances TFIID binding to H3K4me3. Nature.

[41] Bannister A.J., Kouzarides T. (2011). Regulation of chromatin by histone modifications. Cell Res..

[42] Audia J.E., Campbell R.M. (2016). Histone Modifications and Cancer. Cold Spring Harb. Perspect. Biol..

[43] Moresi V., Marroncelli N., Coletti D., Adamo S. (2015). Regulation of skeletal muscle development and homeostasis by gene imprinting, histone acetylation and microRNA. Biochim. Biophys. Acta.

[44] Greer E.L., Shi Y. (2012). Histone methylation: A dynamic mark in health, disease and inheritance. Nat. Rev. Genet..

[45] Tan M., Luo H., Lee S., Jin F., Yang J.S., Montellier E., Buchou T., Cheng Z., Rousseaux S., Rajagopal N. (2011). Identification of 67 histone marks and histone lysine crotonylation as a new type of histone modification. Cell.

[46] Martin C., Zhang Y. (2005). The diverse functions of histone lysine methylation. Nat. Rev. Mol. Cell Biol..

[47] Kouzarides T. (2007). Chromatin modifications and their function. Cell.

[48] Shi Y., Lan F., Matson C., Mulligan P., Whetstine J.R., Cole P.A., Casero R.A., Shi Y. (2004). Histone demethylation mediated by the nuclear amine oxidase homolog LSD1. Cell.

[49] Trievel R.C., Beach B.M., Dirk L.M., Houtz R.L., Hurley J.H. (2002). Structure and catalytic mechanism of a SET domain protein methyltransferase. Cell.

[50] Mal A., Harter M.L. (2003). MyoD is functionally linked to the silencing of a muscle-specific regulatory gene prior to skeletal myogenesis. Proc. Natl. Acad. Sci. USA.

[51] Zhang C.L., McKinsey T.A., Olson E.N. (2002). Association of class II histone deacetylases with heterochromatin protein 1: Potential role for histone methylation in control of muscle differentiation. Mol. Cell Biol..

[52] Mal A.K. (2006). Histone methyltransferase Suv39h1 represses MyoD-stimulated myogenic differentiation. EMBO J..

[53] Ait-Si-Ali S., Guasconi V., Fritsch L., Yahi H., Sekhri R., Naguibneva I., Robin P., Cabon F., Polesskaya A., Harel-Bellan A. (2004). A Suv39h-dependent mechanism for silencing S-phase genes in differentiating but not in cycling cells. EMBO J..

[54] Lee H., Habas R., Abate-Shen C. (2004). MSX1 cooperates with histone H1b for inhibition of transcription and myogenesis. Science.

[55] Ling B.M., Bharathy N., Chung T.K., Kok W.K., Li S., Tan Y.H., Rao V.K., Gopinadhan S., Sartorelli V., Walsh M.J. (2012). Lysine methyltransferase G9a methylates the transcription factor MyoD and regulates skeletal muscle differentiation. Proc. Natl. Acad. Sci. USA.

[56] Choi J., Jang H., Kim H., Kim S.T., Cho E.J., Youn H.D. (2010). Histone demethylase LSD1 is required to induce skeletal muscle differentiation by regulating myogenic factors. Biochem. Biophys. Res. Commun..

[57] Zhu Q., Liang F., Cai S., Luo X., Duo T., Liang Z., He Z., Chen Y., Mo D. (2021). KDM4A regulates myogenesis by demethylating H3K9me3 of myogenic regulatory factors. Cell Death Dis..

[58] Palacios D., Mozzetta C., Consalvi S., Caretti G., Saccone V., Proserpio V., Marquez V.E., Valente S., Mai A., Forcales S.V. (2010). TNF/p38alpha/polycomb signaling to Pax7 locus in satellite cells links inflammation to the epigenetic control of muscle regeneration. Cell Stem Cell.

[59] Peng J.C., Valouev A., Swigut T., Zhang J., Zhao Y., Sidow A., Wysocka J. (2009). Jarid2/Jumonji coordinates control of PRC2 enzymatic activity and target gene occupancy in pluripotent cells. Cell.

[60] Stojic L., Jasencakova Z., Prezioso C., Stutzer A., Bodega B., Pasini D., Klingberg R., Mozzetta C., Margueron R., Puri P.L. (2011). Chromatin regulated interchange between polycomb repressive complex 2 (PRC2)-Ezh2 and PRC2-Ezh1 complexes controls myogenin activation in skeletal muscle cells. Epigenetics Chromatin.

[61] Margueron R., Li G., Sarma K., Blais A., Zavadil J., Woodcock C.L., Dynlacht B.D., Reinberg D. (2008). Ezh1 and Ezh2 maintain repressive chromatin through different mechanisms. Mol. Cell.

[62] Seenundun S., Rampalli S., Liu Q.C., Aziz A., Palii C., Hong S., Blais A., Brand M., Ge K., Dilworth F.J. (2010). UTX mediates demethylation of H3K27me3 at muscle-specific genes during myogenesis. EMBO J..

[63] Boonsanay V., Zhang T., Georgieva A., Kostin S., Qi H., Yuan X., Zhou Y., Braun T. (2016). Regulation of Skeletal Muscle Stem Cell Quiescence by Suv4-20h1-Dependent Facultative Heterochromatin Formation. Cell Stem Cell.

[64] Tao Y., Neppl R.L., Huang Z.P., Chen J., Tang R.H., Cao R., Zhang Y., Jin S.W., Wang D.Z. (2011). The histone methyltransferase Set7/9 promotes myoblast differentiation and myofibril assembly. J. Cell Biol..

[65] McKinnell I.W., Ishibashi J., Le Grand F., Punch V.G., Addicks G.C., Greenblatt J.F., Dilworth F.J., Rudnicki M.A. (2008). Pax7 activates myogenic genes by recruitment of a histone methyltransferase complex. Nat. Cell Biol..

[66] Diao Y., Guo X., Li Y., Sun K., Lu L., Jiang L., Fu X., Zhu H., Sun H., Wang H. (2012). Pax3/7BP is a Pax7- and Pax3-binding protein that regulates the proliferation of muscle precursor cells by an epigenetic mechanism. Cell Stem Cell.

[67] Kawabe Y., Wang Y.X., McKinnell I.W., Bedford M.T., Rudnicki M.A. (2012). Carm1 regulates Pax7 transcriptional activity through MLL1/2 recruitment during asymmetric satellite stem cell divisions. Cell Stem Cell.

[68] Soleimani V.D., Punch V.G., Kawabe Y., Jones A.E., Palidwor G.A., Porter C.J., Cross J.W., Carvajal J.J., Kockx C.E., van IJcken W.F. (2012). Transcriptional dominance of Pax7 in adult myogenesis is due to high-affinity recognition of homeodomain motifs. Dev. Cell.

[69] Rampalli S., Li L., Mak E., Ge K., Brand M., Tapscott S.J., Dilworth F.J. (2007). p38 MAPK signaling regulates recruitment of Ash2L-containing methyltransferase complexes to specific genes during differentiation. Nat. Struct. Mol. Biol..

[70] Sebastian S., Sreenivas P., Sambasivan R., Cheedipudi S., Kandalla P., Pavlath G.K., Dhawan J. (2009). MLL5, a trithorax homolog, indirectly regulates H3K4 methylation, represses cyclin A2 expression, and promotes myogenic differentiation. Proc. Natl. Acad. Sci. USA.

[71] Matteini F., Andresini O., Petrai S., Battistelli C., Rossi M.N., Maione R. (2020). Poly(ADP-ribose) Polymerase 1 (PARP1) restrains MyoD-dependent gene expression during muscle differentiation. Sci. Rep..

[72] Edmunds J.W., Mahadevan L.C., Clayton A.L. (2008). Dynamic histone H3 methylation during gene induction: HYPB/Setd2 mediates all H3K36 trimethylation. EMBO J..

[73] Yi X., Tao Y., Lin X., Dai Y., Yang T., Yue X., Jiang X., Li X., Jiang D.S., Andrade K.C. (2017). Histone methyltransferase Setd2 is critical for the proliferation and differentiation of myoblasts. Biochim. Biophys. Acta Mol. Cell Res..

[74] Rea S., Eisenhaber F., O’Carroll D., Strahl B.D., Sun Z.W., Schmid M., Opravil S., Mechtler K., Ponting C.P., Allis C.D. (2000). Regulation of chromatin structure by site-specific histone H3 methyltransferases. Nature.

[75] Fritsch L., Robin P., Mathieu J.R., Souidi M., Hinaux H., Rougeulle C., Harel-Bellan A., Ameyar-Zazoua M., Ait-Si-Ali S. (2010). A subset of the histone H3 lysine 9 methyltransferases Suv39h1, G9a, GLP, and SETDB1 participate in a multimeric complex. Mol. Cell.

[76] Tachibana M., Sugimoto K., Fukushima T., Shinkai Y. (2001). Set domain-containing protein, G9a, is a novel lysine-preferring mammalian histone methyltransferase with hyperactivity and specific selectivity to lysines 9 and 27 of histone H3. J. Biol. Chem..

[77] Zhang R.H., Judson R.N., Liu D.Y., Kast J., Rossi F.M. (2016). The lysine methyltransferase Ehmt2/G9a is dispensable for skeletal muscle development and regeneration. Skelet. Muscle.

[78] Ling B.M., Gopinadhan S., Kok W.K., Shankar S.R., Gopal P., Bharathy N., Wang Y., Taneja R. (2012). G9a mediates Sharp-1-dependent inhibition of skeletal muscle differentiation. Mol. Biol. Cell.

[79] Verrier L., Escaffit F., Chailleux C., Trouche D., Vandromme M. (2011). A new isoform of the histone demethylase JMJD2A/KDM4A is required for skeletal muscle differentiation. PLoS Genet..

[80] Choi J.H., Song Y.J., Lee H. (2015). The histone demethylase KDM4B interacts with MyoD to regulate myogenic differentiation in C2C12 myoblast cells. Biochem. Biophys. Res. Commun..

[81] Wang H., Cao R., Xia L., Erdjument-Bromage H., Borchers C., Tempst P., Zhang Y. (2001). Purification and functional characterization of a histone H3-lysine 4-specific methyltransferase. Mol. Cell.

[82] Asp P., Blum R., Vethantham V., Parisi F., Micsinai M., Cheng J., Bowman C., Kluger Y., Dynlacht B.D. (2011). Genome-wide remodeling of the epigenetic landscape during myogenic differentiation. Proc. Natl. Acad. Sci. USA.

[83] Dilworth F.J., Blais A. (2011). Epigenetic regulation of satellite cell activation during muscle regeneration. Stem Cell Res. Ther..

[84] Mousavi K., Zare H., Wang A.H., Sartorelli V. (2012). Polycomb protein Ezh1 promotes RNA polymerase II elongation. Mol. Cell.

[85] Harada A., Okada S., Konno D., Odawara J., Yoshimi T., Yoshimura S., Kumamaru H., Saiwai H., Tsubota T., Kurumizaka H. (2012). Chd2 interacts with H3.3 to determine myogenic cell fate. EMBO J..

[86] Harada A., Maehara K., Sato Y., Konno D., Tachibana T., Kimura H., Ohkawa Y. (2015). Incorporation of histone H3.1 suppresses the lineage potential of skeletal muscle. Nucleic Acids Res..

[87] Yang J.H., Song Y., Seol J.H., Park J.Y., Yang Y.J., Han J.W., Youn H.D., Cho E.J. (2011). Myogenic transcriptional activation of MyoD mediated by replication-independent histone deposition. Proc. Natl. Acad. Sci. USA.

[88] Bae S., Lesch B.J. (2020). H3K4me1 Distribution Predicts Transcription State and Poising at Promoters. Front. Cell Dev. Biol..

[89] Ringrose L., Paro R. (2004). Epigenetic regulation of cellular memory by the Polycomb and Trithorax group proteins. Annu. Rev. Genet..

[90] Krishnakumar R., Kraus W.L. (2010). PARP-1 regulates chromatin structure and transcription through a KDM5B-dependent pathway. Mol. Cell.

[91] Minotti R., Andersson A., Hottiger M.O. (2015). ARTD1 Suppresses Interleukin 6 Expression by Repressing MLL1-Dependent Histone H3 Trimethylation. Mol. Cell Biol..

[92] Friedmann D.R., Marmorstein R. (2013). Structure and mechanism of non-histone protein acetyltransferase enzymes. FEBS J..

[93] Elmallah M.I.Y., Micheau O. (2019). Epigenetic Regulation of TRAIL Signaling: Implication for Cancer Therapy. Cancers.

[94] Furdas S.D., Kannan S., Sippl W., Jung M. (2012). Small molecule inhibitors of histone acetyltransferases as epigenetic tools and drug candidates. Arch. Pharm..

[95] Marmorstein R., Zhou M.M. (2014). Writers and readers of histone acetylation: Structure, mechanism, and inhibition. Cold Spring Harb. Perspect. Biol..

[96] Yang X.J., Seto E. (2007). HATs and HDACs: From structure, function and regulation to novel strategies for therapy and prevention. Oncogene.

[97] Seto E., Yoshida M. (2014). Erasers of histone acetylation: The histone deacetylase enzymes. Cold Spring Harb. Perspect. Biol..

[98] Wapenaar H., Dekker F.J. (2016). Histone acetyltransferases: Challenges in targeting bi-substrate enzymes. Clin. Epigenetics.

[99] Doi M., Hirayama J., Sassone-Corsi P. (2006). Circadian regulator CLOCK is a histone acetyltransferase. Cell.

[100] Addicks G.C., Zhang H., Ryu D., Vasam G., Green A.E., Marshall P.L., Patel S., Kang B.E., Kim D., Katsyuba E. (2022). GCN5 maintains muscle integrity by acetylating YY1 to promote dystrophin expression. J. Cell Biol..

[101] Santi S., Cenni V., Capanni C., Lattanzi G., Mattioli E. (2020). PCAF Involvement in Lamin A/C-HDAC2 Interplay during the Early Phase of Muscle Differentiation. Cells.

[102] Kim J.W., Jang S.M., Kim C.H., An J.H., Kang E.J., Choi K.H. (2011). Tip60 regulates myoblast differentiation by enhancing the transcriptional activity of MyoD via their physical interactions. FEBS J..

[103] Eckner R., Yao T.P., Oldread E., Livingston D.M. (1996). Interaction and functional collaboration of p300/CBP and bHLH proteins in muscle and B-cell differentiation. Genes Dev..

[104] Roth J.F., Shikama N., Henzen C., Desbaillets I., Lutz W., Marino S., Wittwer J., Schorle H., Gassmann M., Eckner R. (2003). Differential role of p300 and CBP acetyltransferase during myogenesis: p300 acts upstream of MyoD and Myf5. EMBO J..

[105] Mal A., Sturniolo M., Schiltz R.L., Ghosh M.K., Harter M.L. (2001). A role for histone deacetylase HDAC1 in modulating the transcriptional activity of MyoD: Inhibition of the myogenic program. EMBO J..

[106] Bossone K.A., Ellis J.A., Holaska J.M. (2020). Histone acetyltransferase inhibition rescues differentiation of emerin-deficient myogenic progenitors. Muscle Nerve.

[107] Qian Z., Ye J., Li J., Che Y., Yu W., Xu P., Lin J., Ye F., Xu X., Su Z. (2023). Decrotonylation of AKT1 promotes AKT1 phosphorylation and activation during myogenic differentiation. J. Adv. Res..

[108] Marroncelli N., Bianchi M., Bertin M., Consalvi S., Saccone V., De Bardi M., Puri P.L., Palacios D., Adamo S., Moresi V. (2018). HDAC4 regulates satellite cell proliferation and differentiation by targeting P21 and Sharp1 genes. Sci. Rep..

[109] Renzini A., Marroncelli N., Noviello C., Moresi V., Adamo S. (2018). HDAC4 Regulates Skeletal Muscle Regeneration via Soluble Factors. Front. Physiol..

[110] Haberland M., Arnold M.A., McAnally J., Phan D., Kim Y., Olson E.N. (2007). Regulation of HDAC9 gene expression by MEF2 establishes a negative-feedback loop in the transcriptional circuitry of muscle differentiation. Mol. Cell Biol..

[111] Myers M.J., Shepherd D.L., Durr A.J., Stanton D.S., Mohamed J.S., Hollander J.M., Alway S.E. (2019). The role of SIRT1 in skeletal muscle function and repair of older mice. J. Cachexia Sarcopenia Muscle.

[112] Lee E.J., Lee M.M., Park S., Jeong K.S. (2022). Sirt2 positively regulates muscle regeneration after Notexin-induced muscle injury. Exp. Mol. Pathol..

[113] Lin L., Chen K., Abdel Khalek W., Ward J.L., Yang H., Chabi B., Wrutniak-Cabello C., Tong Q. (2014). Regulation of skeletal muscle oxidative capacity and muscle mass by SIRT3. PLoS ONE.

[114] Byun S.K., An T.H., Son M.J., Lee D.S., Kang H.S., Lee E.W., Han B.S., Kim W.K., Bae K.H., Oh K.J. (2017). HDAC11 Inhibits Myoblast Differentiation through Repression of MyoD-Dependent Transcription. Mol. Cells.

[115] Nunez-Alvarez Y., Hurtado E., Munoz M., Garcia-Tunon I., Rech G.E., Pluvinet R., Sumoy L., Pendas A.M., Peinado M.A., Suelves M. (2021). Loss of HDAC11 accelerates skeletal muscle regeneration in mice. FEBS J..

[116] Ochiai N., Nishizuka M., Osada S., Imagawa M. (2016). Fad24, a Positive Regulator of Adipogenesis, Is Required for S Phase Re-entry of C2C12 Myoblasts Arrested in G0 Phase and Involved in p27(Kip1) Expression at the Protein Level. Biol. Pharm. Bull..

[117] LaBarge S.A., Migdal C.W., Buckner E.H., Okuno H., Gertsman I., Stocks B., Barshop B.A., Nalbandian S.R., Philp A., McCurdy C.E. (2016). p300 is not required for metabolic adaptation to endurance exercise training. FASEB J..

[118] Chen J., Wang Y., Hamed M., Lacroix N., Li Q. (2015). Molecular Basis for the Regulation of Transcriptional Coactivator p300 in Myogenic Differentiation. Sci. Rep..

[119] Joung H., Kwon S., Kim K.H., Lee Y.G., Shin S., Kwon D.H., Lee Y.U., Kook T., Choe N., Kim J.C. (2018). Sumoylation of histone deacetylase 1 regulates MyoD signaling during myogenesis. Exp. Mol. Med..

[120] Ferrari L., Bragato C., Brioschi L., Spreafico M., Esposito S., Pezzotta A., Pizzetti F., Moreno-Fortuny A., Bellipanni G., Giordano A. (2019). HDAC8 regulates canonical Wnt pathway to promote differentiation in skeletal muscles. J. Cell Physiol..

[121] Habibian J.S., Bolino M.J., Ferguson B.S. (2023). HDAC8 regulates protein kinase D phosphorylation in skeletal myoblasts in response to stress signaling. Biochem. Biophys. Res. Commun..

[122] Lu J., McKinsey T.A., Zhang C.L., Olson E.N. (2000). Regulation of skeletal myogenesis by association of the MEF2 transcription factor with class II histone deacetylases. Mol. Cell.

[123] Miska E.A., Karlsson C., Langley E., Nielsen S.J., Pines J., Kouzarides T. (1999). HDAC4 deacetylase associates with and represses the MEF2 transcription factor. EMBO J..

[124] McKinsey T.A., Zhang C.L., Lu J., Olson E.N. (2000). Signal-dependent nuclear export of a histone deacetylase regulates muscle differentiation. Nature.

[125] Zhao J., Shen X., Cao X., He H., Han S., Chen Y., Cui C., Wei Y., Wang Y., Li D. (2020). HDAC4 Regulates the Proliferation, Differentiation and Apoptosis of Chicken Skeletal Muscle Satellite Cells. Animals.

[126] Han Z., Chang C., Zhu W., Zhang Y., Zheng J., Kang X., Jin G., Gong Z. (2021). Role of SIRT2 in regulating the dexamethasone-activated autophagy pathway in skeletal muscle atrophy. Biochem. Cell Biol..

[127] Song M.Y., Han C.Y., Moon Y.J., Lee J.H., Bae E.J., Park B.H. (2022). Sirt6 reprograms myofibers to oxidative type through CREB-dependent Sox6 suppression. Nat. Commun..

[128] Zhang R., Pan Y., Feng W., Zhao Y., Yang Y., Wang L., Zhang Y., Cheng J., Jiang Q., Zheng Z. (2022). HDAC11 Regulates the Proliferation of Bovine Muscle Stem Cells through the Notch Signaling Pathway and Inhibits Muscle Regeneration. J. Agric. Food Chem..

[129] Zhang D., Tang Z., Huang H., Zhou G., Cui C., Weng Y., Liu W., Kim S., Lee S., Perez-Neut M. (2019). Metabolic regulation of gene expression by histone lactylation. Nature.

[130] Xie Y., Hu H., Liu M., Zhou T., Cheng X., Huang W., Cao L. (2022). The role and mechanism of histone lactylation in health and diseases. Front. Genet..

[131] Ohno Y., Ando K., Ito T., Suda Y., Matsui Y., Oyama A., Kaneko H., Yokoyama S., Egawa T., Goto K. (2019). Lactate Stimulates a Potential for Hypertrophy and Regeneration of Mouse Skeletal Muscle. Nutrients.

[132] Tsukamoto S., Shibasaki A., Naka A., Saito H., Iida K. (2018). Lactate Promotes Myoblast Differentiation and Myotube Hypertrophy via a Pathway Involving MyoD In Vitro and Enhances Muscle Regeneration In Vivo. Int. J. Mol. Sci..

[133] Willkomm L., Schubert S., Jung R., Elsen M., Borde J., Gehlert S., Suhr F., Bloch W. (2014). Lactate regulates myogenesis in C2C12 myoblasts in vitro. Stem Cell Res..

[134] Galle E., Wong C.W., Ghosh A., Desgeorges T., Melrose K., Hinte L.C., Castellano-Castillo D., Engl M., de Sousa J.A., Ruiz-Ojeda F.J. (2022). H3K18 lactylation marks tissue-specific active enhancers. Genome Biol..

[135] Dai W., Wu G., Liu K., Chen Q., Tao J., Liu H., Shen M. (2023). Lactate promotes myogenesis via activating H3K9 lactylation-dependent up-regulation of Neu2 expression. J. Cachexia Sarcopenia Muscle.

[136] Desgeorges T., Galle E., Zhang J., von Meyenn F., De Bock K. (2024). Histone lactylation in macrophages is predictive for gene expression changes during ischemia induced-muscle regeneration. Mol. Metab..

[137] Yoshihara T., Machida S., Tsuzuki T., Kakigi R., Chang S.W., Sugiura T., Naito H. (2019). Age-related changes in histone modification in rat gastrocnemius muscle. Exp. Gerontol..

[138] Kawano F., Nimura K., Ishino S., Nakai N., Nakata K., Ohira Y. (2015). Differences in histone modifications between slow- and fast-twitch muscle of adult rats and following overload, denervation, or valproic acid administration. J. Appl. Physiol..

[139] Ryder D.J., Judge S.M., Beharry A.W., Farnsworth C.L., Silva J.C., Judge A.R. (2015). Identification of the Acetylation and Ubiquitin-Modified Proteome during the Progression of Skeletal Muscle Atrophy. PLoS ONE.

[140] Liang W., Xu F., Li L., Peng C., Sun H., Qiu J., Sun J. (2024). Epigenetic control of skeletal muscle atrophy. Cell Mol. Biol. Lett..

[141] Acharyya S., Sharma S.M., Cheng A.S., Ladner K.J., He W., Kline W., Wang H., Ostrowski M.C., Huang T.H., Guttridge D.C. (2010). TNF inhibits Notch-1 in skeletal muscle cells by Ezh2 and DNA methylation mediated repression: Implications in duchenne muscular dystrophy. PLoS ONE.

[142] Acharyya S., Villalta S.A., Bakkar N., Bupha-Intr T., Janssen P.M., Carathers M., Li Z.W., Beg A.A., Ghosh S., Sahenk Z. (2007). Interplay of IKK/NF-kappaB signaling in macrophages and myofibers promotes muscle degeneration in Duchenne muscular dystrophy. J. Clin. Investig..

[143] Lu X., Liang B., Li S., Chen Z., Chang W. (2020). Modulation of HOXA9 after skeletal muscle denervation and reinnervation. Am. J. Physiol. Cell Physiol..

[144] Proserpio V., Fittipaldi R., Ryall J.G., Sartorelli V., Caretti G. (2013). The methyltransferase SMYD3 mediates the recruitment of transcriptional cofactors at the myostatin and c-Met genes and regulates skeletal muscle atrophy. Genes Dev..

[145] Sakaguchi M., Cai W., Wang C.H., Cederquist C.T., Damasio M., Homan E.P., Batista T., Ramirez A.K., Gupta M.K., Steger M. (2019). FoxK1 and FoxK2 in insulin regulation of cellular and mitochondrial metabolism. Nat. Commun..

[146] Senf S.M., Sandesara P.B., Reed S.A., Judge A.R. (2011). p300 Acetyltransferase activity differentially regulates the localization and activity of the FOXO homologues in skeletal muscle. Am. J. Physiol. Cell Physiol..

[147] Bertaggia E., Coletto L., Sandri M. (2012). Posttranslational modifications control FoxO3 activity during denervation. Am. J. Physiol. Cell Physiol..

[148] Sandri M., Sandri C., Gilbert A., Skurk C., Calabria E., Picard A., Walsh K., Schiaffino S., Lecker S.H., Goldberg A.L. (2004). Foxo transcription factors induce the atrophy-related ubiquitin ligase atrogin-1 and cause skeletal muscle atrophy. Cell.

[149] Chamberlain W., Gonnella P., Alamdari N., Aversa Z., Hasselgren P.O. (2012). Multiple muscle wasting-related transcription factors are acetylated in dexamethasone-treated muscle cells. Biochem. Cell Biol..

[150] Sin T.K., Zhu J.Z., Zhang G., Li Y.P. (2019). p300 Mediates Muscle Wasting in Lewis Lung Carcinoma. Cancer Res..

[151] Zhang G., Jin B., Li Y.P. (2011). C/EBPbeta mediates tumour-induced ubiquitin ligase atrogin1/MAFbx upregulation and muscle wasting. EMBO J..

[152] Fan Z., Wu J., Chen Q.N., Lyu A.K., Chen J.L., Sun Y., Lyu Q., Zhao Y.X., Guo A., Liao Z.Y. (2020). Type 2 diabetes-induced overactivation of P300 contributes to skeletal muscle atrophy by inhibiting autophagic flux. Life Sci..

[153] Sin T.K., Zhang G., Zhang Z., Zhu J.Z., Zuo Y., Frost J.A., Li M., Li Y.P. (2021). Cancer-Induced Muscle Wasting Requires p38beta MAPK Activation of p300. Cancer Res..

[154] Beharry A.W., Sandesara P.B., Roberts B.M., Ferreira L.F., Senf S.M., Judge A.R. (2014). HDAC1 activates FoxO and is both sufficient and required for skeletal muscle atrophy. J. Cell Sci..

[155] Cacchiarelli D., Martone J., Girardi E., Cesana M., Incitti T., Morlando M., Nicoletti C., Santini T., Sthandier O., Barberi L. (2010). MicroRNAs involved in molecular circuitries relevant for the Duchenne muscular dystrophy pathogenesis are controlled by the dystrophin/nNOS pathway. Cell Metab..

[156] Colussi C., Mozzetta C., Gurtner A., Illi B., Rosati J., Straino S., Ragone G., Pescatori M., Zaccagnini G., Antonini A. (2008). HDAC2 blockade by nitric oxide and histone deacetylase inhibitors reveals a common target in Duchenne muscular dystrophy treatment. Proc. Natl. Acad. Sci. USA.

[157] Spreafico M., Cafora M., Bragato C., Capitanio D., Marasca F., Bodega B., De Palma C., Mora M., Gelfi C., Marozzi A. (2021). Targeting HDAC8 to ameliorate skeletal muscle differentiation in Duchenne muscular dystrophy. Pharmacol. Res..

[158] Berdeaux R., Goebel N., Banaszynski L., Takemori H., Wandless T., Shelton G.D., Montminy M. (2007). SIK1 is a class II HDAC kinase that promotes survival of skeletal myocytes. Nat. Med..

[159] Cohen T.J., Waddell D.S., Barrientos T., Lu Z., Feng G., Cox G.A., Bodine S.C., Yao T.P. (2007). The histone deacetylase HDAC4 connects neural activity to muscle transcriptional reprogramming. J. Biol. Chem..

[160] Pigna E., Simonazzi E., Sanna K., Bernadzki K.M., Proszynski T., Heil C., Palacios D., Adamo S., Moresi V. (2019). Histone deacetylase 4 protects from denervation and skeletal muscle atrophy in a murine model of amyotrophic lateral sclerosis. EBioMedicine.

[161] Moresi V., Williams A.H., Meadows E., Flynn J.M., Potthoff M.J., McAnally J., Shelton J.M., Backs J., Klein W.H., Richardson J.A. (2010). Myogenin and class II HDACs control neurogenic muscle atrophy by inducing E3 ubiquitin ligases. Cell.

[162] Zhang Z., Zhang L., Zhou Y., Li L., Zhao J., Qin W., Jin Z., Liu W. (2019). Increase in HDAC9 suppresses myoblast differentiation via epigenetic regulation of autophagy in hypoxia. Cell Death Dis..

[163] Osseni A., Ravel-Chapuis A., Belotti E., Scionti I., Gangloff Y.G., Moncollin V., Mazelin L., Mounier R., Leblanc P., Jasmin B.J. (2022). Pharmacological inhibition of HDAC6 improves muscle phenotypes in dystrophin-deficient mice by downregulating TGF-beta via Smad3 acetylation. Nat. Commun..

[164] Surinlert P., Thitiphatphuvanon T., Khimmaktong W., Pholpramoo C., Tipbunjong C. (2021). Hyperglycemia induced C2C12 myoblast cell cycle arrest and skeletal muscle atrophy by modulating sirtuins gene expression in rats. Pol. J. Vet. Sci..

[165] Lee H., Kim S.Y., Lim Y. (2023). Lespedeza bicolor extract supplementation reduced hyperglycemia-induced skeletal muscle damage by regulation of AMPK/SIRT/PGC1alpha-related energy metabolism in type 2 diabetic mice. Nutr. Res..

[166] Lee D., Goldberg A.L. (2013). SIRT1 protein, by blocking the activities of transcription factors FoxO1 and FoxO3, inhibits muscle atrophy and promotes muscle growth. J. Biol. Chem..

[167] Dugdale H.F., Hughes D.C., Allan R., Deane C.S., Coxon C.R., Morton J.P., Stewart C.E., Sharples A.P. (2018). The role of resveratrol on skeletal muscle cell differentiation and myotube hypertrophy during glucose restriction. Mol. Cell Biochem..

[168] Chalkiadaki A., Igarashi M., Nasamu A.S., Knezevic J., Guarente L. (2014). Muscle-specific SIRT1 gain-of-function increases slow-twitch fibers and ameliorates pathophysiology in a mouse model of duchenne muscular dystrophy. PLoS Genet..

[169] Simionescu-Bankston A., Kumar A. (2016). Noncoding RNAs in the regulation of skeletal muscle biology in health and disease. J. Mol. Med..

[170] Lee S.J., Huynh T.V., Lee Y.S., Sebald S.M., Wilcox-Adelman S.A., Iwamori N., Lepper C., Matzuk M.M., Fan C.M. (2012). Role of satellite cells versus myofibers in muscle hypertrophy induced by inhibition of the myostatin/activin signaling pathway. Proc. Natl. Acad. Sci. USA.

[171] Hitachi K., Tsuchida K. (2013). Role of microRNAs in skeletal muscle hypertrophy. Front. Physiol..

[172] McGee S.L., Fairlie E., Garnham A.P., Hargreaves M. (2009). Exercise-induced histone modifications in human skeletal muscle. J. Physiol..

[173] Thalacker-Mercer A., Stec M., Cui X., Cross J., Windham S., Bamman M. (2013). Cluster analysis reveals differential transcript profiles associated with resistance training-induced human skeletal muscle hypertrophy. Physiol. Genomics.

[174] Potthoff M.J., Wu H., Arnold M.A., Shelton J.M., Backs J., McAnally J., Richardson J.A., Bassel-Duby R., Olson E.N. (2007). Histone deacetylase degradation and MEF2 activation promote the formation of slow-twitch myofibers. J. Clin. Investig..

[175] Chabert C., Khochbin S., Rousseaux S., Furze R., Smithers N., Prinjha R., Schlattner U., Pison C., Dubouchaud H. (2017). Muscle hypertrophy in hypoxia with inflammation is controlled by bromodomain and extra-terminal domain proteins. Sci. Rep..

[176] Moretti I., Ciciliot S., Dyar K.A., Abraham R., Murgia M., Agatea L., Akimoto T., Bicciato S., Forcato M., Pierre P. (2016). MRF4 negatively regulates adult skeletal muscle growth by repressing MEF2 activity. Nat. Commun..

[177] Winbanks C.E., Beyer C., Hagg A., Qian H., Sepulveda P.V., Gregorevic P. (2013). miR-206 represses hypertrophy of myogenic cells but not muscle fibers via inhibition of HDAC4. PLoS ONE.

[178] Koltai E., Bori Z., Chabert C., Dubouchaud H., Naito H., Machida S., Davies K.J., Murlasits Z., Fry A.C., Boldogh I. (2017). SIRT1 may play a crucial role in overload-induced hypertrophy of skeletal muscle. J. Physiol..

[179] Khurana T.S., Hoffman E.P., Kunkel L.M. (1990). Identification of a chromosome 6-encoded dystrophin-related protein. J. Biol. Chem..

[180] Guiraud S., Aartsma-Rus A., Vieira N.M., Davies K.E., van Ommen G.J., Kunkel L.M. (2015). The Pathogenesis and Therapy of Muscular Dystrophies. Annu. Rev. Genomics Hum. Genet..

[181] Colussi C., Gurtner A., Rosati J., Illi B., Ragone G., Piaggio G., Moggio M., Lamperti C., D’Angelo G., Clementi E. (2009). Nitric oxide deficiency determines global chromatin changes in Duchenne muscular dystrophy. FASEB J..

[182] Consalvi S., Saccone V., Mozzetta C. (2014). Histone deacetylase inhibitors: A potential epigenetic treatment for Duchenne muscular dystrophy. Epigenomics.

[183] Sandona M., Cavioli G., Renzini A., Cedola A., Gigli G., Coletti D., McKinsey T.A., Moresi V., Saccone V. (2023). Histone Deacetylases: Molecular Mechanisms and Therapeutic Implications for Muscular Dystrophies. Int. J. Mol. Sci..

[184] Georgieva A.M., Guo X., Bartkuhn M., Gunther S., Kunne C., Smolka C., Atzberger A., Gartner U., Mamchaoui K., Bober E. (2022). Inactivation of Sirt6 ameliorates muscular dystrophy in mdx mice by releasing suppression of utrophin expression. Nat. Commun..

[185] Renzini A., Marroncelli N., Cavioli G., Di Francescantonio S., Forcina L., Lambridis A., Di Giorgio E., Valente S., Mai A., Brancolini C. (2022). Cytoplasmic HDAC4 regulates the membrane repair mechanism in Duchenne muscular dystrophy. J. Cachexia Sarcopenia Muscle.

[186] (2024). Givinostat (Duvyzat) for Duchenne muscular dystrophy. Med. Lett. Drugs Ther..

[187] Tonini M.M., Passos-Bueno M.R., Cerqueira A., Matioli S.R., Pavanello R., Zatz M. (2004). Asymptomatic carriers and gender differences in facioscapulohumeral muscular dystrophy (FSHD). Neuromuscul. Disord..

[188] Lemmers R.J., van der Vliet P.J., Klooster R., Sacconi S., Camano P., Dauwerse J.G., Snider L., Straasheijm K.R., van Ommen G.J., Padberg G.W. (2010). A unifying genetic model for facioscapulohumeral muscular dystrophy. Science.

[189] Zeng W., de Greef J.C., Chen Y.Y., Chien R., Kong X., Gregson H.C., Winokur S.T., Pyle A., Robertson K.D., Schmiesing J.A. (2009). Specific loss of histone H3 lysine 9 trimethylation and HP1gamma/cohesin binding at D4Z4 repeats is associated with facioscapulohumeral dystrophy (FSHD). PLoS Genet..

[190] Jiang G., Yang F., van Overveld P.G., Vedanarayanan V., van der Maarel S., Ehrlich M. (2003). Testing the position-effect variegation hypothesis for facioscapulohumeral muscular dystrophy by analysis of histone modification and gene expression in subtelomeric 4q. Hum. Mol. Genet..

[191] Haynes P., Bomsztyk K., Miller D.G. (2018). Sporadic DUX4 expression in FSHD myocytes is associated with incomplete repression by the PRC2 complex and gain of H3K9 acetylation on the contracted D4Z4 allele. Epigenetics Chromatin.

[192] Strafella C., Caputo V., Bortolani S., Torchia E., Megalizzi D., Trastulli G., Monforte M., Colantoni L., Caltagirone C., Ricci E. (2023). Whole exome sequencing highlights rare variants in CTCF, DNMT1, DNMT3A, EZH2 and SUV39H1 as associated with FSHD. Front. Genet..

[193] Choi S.H., Gearhart M.D., Cui Z., Bosnakovski D., Kim M., Schennum N., Kyba M. (2016). DUX4 recruits p300/CBP through its C-terminus and induces global H3K27 acetylation changes. Nucleic Acids Res..

[194] Bosnakovski D., da Silva M.T., Sunny S.T., Ener E.T., Toso E.A., Yuan C., Cui Z., Walters M.A., Jadhav A., Kyba M. (2019). A novel P300 inhibitor reverses DUX4-mediated global histone H3 hyperacetylation, target gene expression, and cell death. Sci. Adv..

[195] Ma Z., Chen L., Wang Y., Zhang S., Zheng J., Luo Y., Wang C., Zeng H., Xue L., Tan Z. (2023). Novel insights of EZH2-mediated epigenetic modifications in degenerative musculoskeletal diseases. Ageing Res. Rev..

[196] Saul D., Kosinsky R.L. (2021). Epigenetics of Aging and Aging-Associated Diseases. Int. J. Mol. Sci..

[197] Molinari S., Imbriano C., Moresi V., Renzini A., Belluti S., Lozanoska-Ochser B., Gigli G., Cedola A. (2023). Histone deacetylase functions and therapeutic implications for adult skeletal muscle metabolism. Front. Mol. Biosci..

[198] Setiawan T., Sari I.N., Wijaya Y.T., Julianto N.M., Muhammad J.A., Lee H., Chae J.H., Kwon H.Y. (2023). Cancer cachexia: Molecular mechanisms and treatment strategies. J. Hematol. Oncol..

[199] Zhou L., Zhang T., Shao W., Lu R., Wang L., Liu H., Jiang B., Li S., Zhuo H., Wang S. (2021). Amiloride ameliorates muscle wasting in cancer cachexia through inhibiting tumor-derived exosome release. Skelet. Muscle.

[200] Xu K., Wang Y., Wang F., Guo Y., Ren Y., Low V., Cho S., Liu Q., Qiu Y., Li X. (2025). SIRT6 Ameliorates Cancer Cachexia-Associated Adipose Wasting by Suppressing TNFR2 Signalling in Mice. J. Cachexia Sarcopenia Muscle.

[201] Liu D., Wei B., Liang L., Sheng Y., Sun S., Sun X., Li M., Li H., Yang C., Peng Y. (2024). The Circadian Clock Component RORA Increases Immunosurveillance in Melanoma by Inhibiting PD-L1 Expression. Cancer Res..

[202] Sincennes M.C., Brun C.E., Rudnicki M.A. (2016). Concise Review: Epigenetic Regulation of Myogenesis in Health and Disease. Stem Cells Transl. Med..

[203] Bianchi M., Renzini A., Adamo S., Moresi V. (2017). Coordinated Actions of MicroRNAs with other Epigenetic Factors Regulate Skeletal Muscle Development and Adaptation. Int. J. Mol. Sci..

[204] Yang Y., Fan X., Yan J., Chen M., Zhu M., Tang Y., Liu S., Tang Z. (2021). A comprehensive epigenome atlas reveals DNA methylation regulating skeletal muscle development. Nucleic Acids Res..

[205] Wang Z., Yao H., Lin S., Zhu X., Shen Z., Lu G., Poon W.S., Xie D., Lin M.C., Kung H.F. (2013). Transcriptional and epigenetic regulation of human microRNAs. Cancer Lett..

[206] Szczepanek J., Tretyn A. (2023). MicroRNA-Mediated Regulation of Histone-Modifying Enzymes in Cancer: Mechanisms and Therapeutic Implications. Biomolecules.

[207] Nawaz K., Cziesielski M.J., Mariappan K.G., Cui G., Aranda M. (2022). Histone modifications and DNA methylation act cooperatively in regulating symbiosis genes in the sea anemone Aiptasia. BMC Biol..

[208] Dubois V., Laurent M., Boonen S., Vanderschueren D., Claessens F. (2012). Androgens and skeletal muscle: Cellular and molecular action mechanisms underlying the anabolic actions. Cell Mol. Life Sci..

[209] Nuccio A., Nogueira-Ferreira R., Moreira-Pais A., Attanzio A., Duarte J.A., Luparello C., Ferreira R. (2024). The contribution of mitochondria to age-related skeletal muscle wasting: A sex-specific perspective. Life Sci..

[210] Pataky M.W., Dasari S., Michie K.L., Sevits K.J., Kumar A.A., Klaus K.A., Heppelmann C.J., Robinson M.M., Carter R.E., Lanza I.R. (2023). Impact of biological sex and sex hormones on molecular signatures of skeletal muscle at rest and in response to distinct exercise training modes. Cell Metab..

[211] Urban R.J., Bodenburg Y.H., Gilkison C., Foxworth J., Coggan A.R., Wolfe R.R., Ferrando A. (1995). Testosterone administration to elderly men increases skeletal muscle strength and protein synthesis. Am. J. Physiol..

[212] Pagliarini V., Guerra M., Di Rosa V., Compagnucci C., Sette C. (2020). Combined treatment with the histone deacetylase inhibitor LBH589 and a splice-switch antisense oligonucleotide enhances SMN2 splicing and SMN expression in Spinal Muscular Atrophy cells. J. Neurochem..

